# Preclinical Immunogenicity of a 6-Valent GBS Glycoconjugate Vaccine from a Repeat-Dose GLP Toxicology Study

**DOI:** 10.3390/vaccines13090952

**Published:** 2025-09-05

**Authors:** Aakriti Bajracharya, Gowri Chellappan, Florence Seal, Yutai Zhao, Giriraj Chalke, Neza Chowdhury, Harshita Seth, Jen Gan, Shangdong Guo, Kevin Pinder, Fong Chang, Drew Huff, Abby Mydland, Chloe Wright, Lais Conceicao, Winston Balasundaram, Rama Raghunandan, Anup Datta, Subhash V. Kapre

**Affiliations:** 1 Bacterial Research and Development, Inventprise, Inc., Redmond, WA 98052, USA; 2 Immunology, Manufacturing Sciences and Technology, Inventprise, Inc., Redmond, WA 98052, USA; 3 Quality Control/Micro, Inventprise, Inc., Redmond, WA 98052, USA; 4 Center for Vaccine Innovation and Access (CVIA), Program for Appropriate Technology in Health (PATH), Seattle, WA 98103, USA; 5 Inventprise, Inc., Redmond, WA 98052, USA

**Keywords:** GBS, meningitis, neonates, maternal immunization, conjugate vaccine, rCRM197, HZ-PEG-HZ linker, IgG

## Abstract

**Background/Objectives**: Group B Streptococcus (GBS) is a significant cause of perinatal infection in neonates and infants. Complications could include neonatal sepsis and meningitis, preterm birth, stillbirth, or death. Though no GBS vaccine is currently licensed, maternal immunization is expected to be a highly effective strategy to address invasive GBS disease—particularly in low- and middle-income countries (LMICs), where the disease burden is the greatest and access to existing interventions is limited. In this study, we present a novel hexavalent GBS vaccine candidate with a unique combination of serotypes (ST)—Ia, Ib, II, III, V, and VII—that could be an efficacious and cost-effective intervention, with the broadest coverage of 99% against circulating serotypes globally. **Methods**: The 6-valent conjugate vaccine candidate, GBS-06, is developed using a novel approach by linking the six polysaccharides (PS) to recombinant cross-reactive material 197 (rCRM197) carrier protein derivatized with a hydrazide-polyethylene glycol-hydrazide (HZ-PEG-HZ) linker. A repeat-dose GLP toxicology study with GBS-06 was conducted at the highest clinical dose of 20 µg in rabbits with saline as the placebo control. **Results**: The results reveal induction of robust anti-capsular polysaccharide-specific IgG responses against each of the six serotypes after each dose with the highest antibody GMCs at Day 49 following the third dose. **Conclusions**: Hence, this work is the first demonstration of strong immunogenicity achieved using a linker (HZ-PEG-HZ) for GBS glycoconjugate vaccine development. The positive data from the study have strong implications in the advancement of the candidate for evaluation in clinical trials and provide a licensure pathway for maternal immunization.

## 1. Introduction

GBS or *Streptococcus agalactiae* is a Gram-positive bacterium that is responsible for a serious invasive disease: neonatal sepsis and meningitis [1,2]. GBS infection in newborns is classified into two categories: early-onset disease (EOD), occurring within the first 6 days of birth, and late-onset disease (LOD), occurring between 7 and 89 days after birth [3]. EOD occurs due to transmission of GBS from the mother to the newborn during pregnancy or at birth. Although the nature of transmission of LOD is not completely understood, it is suspected to be attributed to GBS gastrointestinal colonization at birth, breastfeeding, or other external factors [4,5,6].

GBS infections are attributed to a median of 518,000 [uncertainty interval: 36,000–1,142,000] preterm births and more than 90,000 infant fatalities every year, with a 3-fold higher mean fatality rate in the LMICs compared to that of high-income or developed countries [7,8]. More than 40% of overall childhood mortality in LMICs occurs during the neonatal period—most of it in the first week of life. Overall, prevalence of Group B Streptococcus colonization in mothers in South Asian and African countries is approximately 24% [9]. While there has been a decline in infant deaths, the incidence of maternal and neonatal mortality and stillbirth is still very high, mostly in sub-Saharan Africa and South Asia [3]. Testing for GBS during pregnancy has become a standard practice in some developed countries, along with the intravenous administration of intrapartum antibiotic prophylaxis (IAP) during labor. However, in LMICs, there are no programmatic microbiological screening protocols or standardized IAP practices available. Furthermore, IAP administration led to a reduction in EOD but has shown no effect on LOD [4,10]. In this regard, intervention strategies such as maternal vaccination gained great impetus based on the success of other maternal vaccination programs, such as influenza, RSV, and Tdap, that can prevent diseases in newborns [11]. According to the WHO report, vaccination against GBS is an effective, equitable, and affordable approach for eliminating the global disease burden by integration into routine maternal immunization programs [8]. In relation to this, capsular polysaccharides which are implicated in GBS disease virulence can serve as attractive candidates for vaccine development. There are 10 known serotypes of GBS (Ia, Ib, and II-IX) based on the structure of the capsular polysaccharides, with the key antigenic epitope being the terminal sialic acid in the polysaccharide repeating unit [12]. Sialic acid plays a critical role in GBS CPS antigenicity, and antibodies against capsular polysaccharide epitopes have been shown to confer natural immunity against GBS [13]. Further, seminal work by Carol Baker and colleagues firmly established protection against GBS III disease due to the presence of high concentrations of anti-capsular PS III-specific IgG antibodies in maternal sera [14]. These findings in fact laid the cornerstone for GBS polysaccharide-protein conjugate vaccine development wherein vertically transferred immunity through maternal vaccination can confer protection in newborns against GBS infection [15]. In subsequent years, GBS vaccine development efforts gained more momentum globally and were identified as a priority by the WHO in 2015. According to the 2021 WHO assessment report, GBS vaccine with a coverage of at least 90% of the disease-causing serotypes in target regions and 80% efficacy against invasive disease in offsprings is preferred [8]. Hence, a maternal vaccine with 80% efficacy and 90% coverage could prevent more than 200,000 neonatal and maternal GBS cases, and more than 40,000 stillbirths and close to 65,000 infant deaths annually [3].

GBS vaccine development efforts in the last two decades have taken advantage of polysaccharide-protein conjugation technology proven to be effective in vaccines against other encapsulated bacteria, such as *H. influenzae*, *S. pneumoniae*, and *N. meningitidis*. There is no currently approved GBS vaccine, although a few vaccine candidates based on CPS-rCRM197 glycoconjugates or protein subunits are in clinical stages of development [16]. In relation to this, encouraging immunogenicity data have been reported from phase II clinical trial studies of a 6-valent GBS conjugate vaccine covering STs Ia, Ib, II, III, IV, and V in pregnant women [17]. In parallel, vaccines based on the conserved GBS cell-surface Alpha-like proteins AlpCN and RibN or Alp-1N and Alp2/3 N have been evaluated in phase I clinical trials [18] and in phase 2 clinical trials involving pregnant women (NCT05154578).

In this paper, we present preclinical studies of a 6-valent GBS glycoconjugate vaccine, GBS-06, containing the six capsular serotype (ST) PS- Ia, Ib, II, III, V, and VII with rCRM197 as the carrier protein. In particular, including serotype VII—reported in GBS-positive pregnant women in India and Bangladesh—is a distinctive approach that is reported for the first time to our knowledge [19,20]. Although, STs VI-IX relate only to 3% of the clinical isolates from maternal colonization worldwide, there has been a higher prevalence (>10%) of these serotypes in different parts of Asia [21,22].

The 6-valent conjugate vaccine candidate is developed by conjugation of GBS capsular polysaccharide (CPS) to rCRM197 carrier protein derivatized with a HZ-PEG-HZ linker. Some of the advantages of using PEG spacers include literature data on the enhancement of immune responses to vaccines by increasing conjugate half-life, and prevention of steric hindrance, which is important in the context of glycoconjugates involving complex polysaccharide structures and bulky protein groups [23]. Further, PEG-linkers can promote stability by preventing aggregation of biomolecules in solution [24]. Herein, we report the serology data from a GLP toxicology study in a rabbit animal model aimed at assessing the safety and immunogenicity of the GBS-06 vaccine candidate with the potential to be approved as a vaccine for maternal immunization. Top-line results indicate generation of robust immune responses against all GBS serotypes (Ia, Ib, II, III, V, and VII) with increasing IgG concentrations after each dose of GBS-06 vaccine in a three-dose regimen in rabbits.

## 2. Materials and Methods

### 2.1. Animal Ethics Statement

The study was performed at Bioneeds India Private Limited, Karnataka, India in accordance with the recommendation of the Committee for the Control and Supervision of Experiments on Animals (CCSEA) guidelines for the laboratory animal facility, 2018. The study protocol was approved by the Institutional Animal Ethics Committee (IAEC) (Protocol No.: BIO-IAEC-4669, Approval Date: 9 September 2023). The New Zealand white rabbits were procured from Invivo Biosciences (Bengaluru, KA, India).

### 2.2. Capsular Polysaccharide Production

The strains for GBS CPS serotypes were procured from ATCC, Manassas, VA, USA or SSI, Hillerød, Herredsvejen, Denmark. The species and serotype identity were confirmed using DNA sequencing (Accugenix ID-BacSeq, Charles River Laboratories, Wilmington, MA, USA) and the ImmuLexTM Strep-B latex agglutination test (SSI Diagnostica, Hillerød, Herredsvejen, Denmark), respectively.

The fermentation of the GBS bacterial cultures for capsular PS production was performed in yeast extract feed media in a glass fermenter. OD600 was measured at regular intervals for monitoring cell growth and density. During the fermentation run, the pH, temperature, and dissolved oxygen% were maintained at 7.3, 37.0 °C, and >25%, respectively. After termination of the fermentation by base addition, the crude inactivated culture broth was neutralized to pH 7.5 and centrifuged at 15,500× *g* for 25 min followed by diafiltration with water using a 10 kDa cut-off cassette (Repligen Corp., Waltham, MA, USA), maintaining a transmembrane pressure (TMP) of 15 psi. CPS was then subjected to alkaline hydrolysis for removal of protein and nucleic acid impurities, followed by re-N-acetylation of the CPS with equal volumes of acetic anhydride: ethanol mixture (Millipore Sigma, St. Louis, MS, USA) at pH 9.0. In the final polishing step involving hydrophobic interaction chromatography, the PS dissolved in aqueous buffer containing 3 M NaCl, pH 6.8, was loaded in the pre-packed HIC column (Repligen Corp., Waltham, MA, USA) and collected in the flow-through. The product was purified by diafiltration in water, filtered with a 0.22 µm polyethersulfone filter (Thermo Fisher Scientific, Waltham, MA, USA) and stored at −80 °C. The CPS concentration was determined by resorcinol assay [25] and the structural identity was confirmed by NMR.

### 2.3. Conjugation of CPS to rCRM197

The strain of the rCRM197 carrier protein expressed in *E. coli* (EcoCRM^®^) was obtained from Fina Biosolutions, LLC, Rockville, MD, USA. The culture was fermented under controlled conditions maintaining the pH, dissolved oxygen, and temperature, followed by cell lysis and purification. The purity of rCRM197 was analyzed by SEC-HPLC.

As a first step in conjugation, the purified rCRM197 was derivatized with HZ-PEG-HZ linker, 1 kDa (Hunan Huateng Pharmaceutical Co., Ltd., Changsha City, Hunan Province, China), following previously described methods [26]. Briefly, HZ-PEG-HZ linker dissolved in MES buffer (Millipore Sigma, St. Louis, MO, USA) was added to the rCRM197 carrier protein at the desired input ratios in a glass vessel (AGI Inc., Austin, TX, USA) provided with software controls for pH, stirring, and temperature. 1-Ethyl-3-(3-dimethylaminopropyl) carbodiimide (EDC) (Millipore Sigma, St. Louis, MO, USA) was dissolved in MES buffer and added to rCRM197-linker solution at the desired ratios. Following the derivatization process, the product was purified by diafiltration using a 30 kDa cassette (Sartorius Stedim, Göttingen, Germany) and filtered using a 0.22 µm filter (Thermo Fisher Scientific, Waltham, MA, USA). Protein concentration was estimated using the A280 method using the extinction coefficient of 0.934 mg^−1^ mL cm^−1^. The degree of derivatization was determined by established 2, 4, and 6-Trinitrobenzene sulfonic acid (TNBS) assay protocol [27].

**Figure 1:**
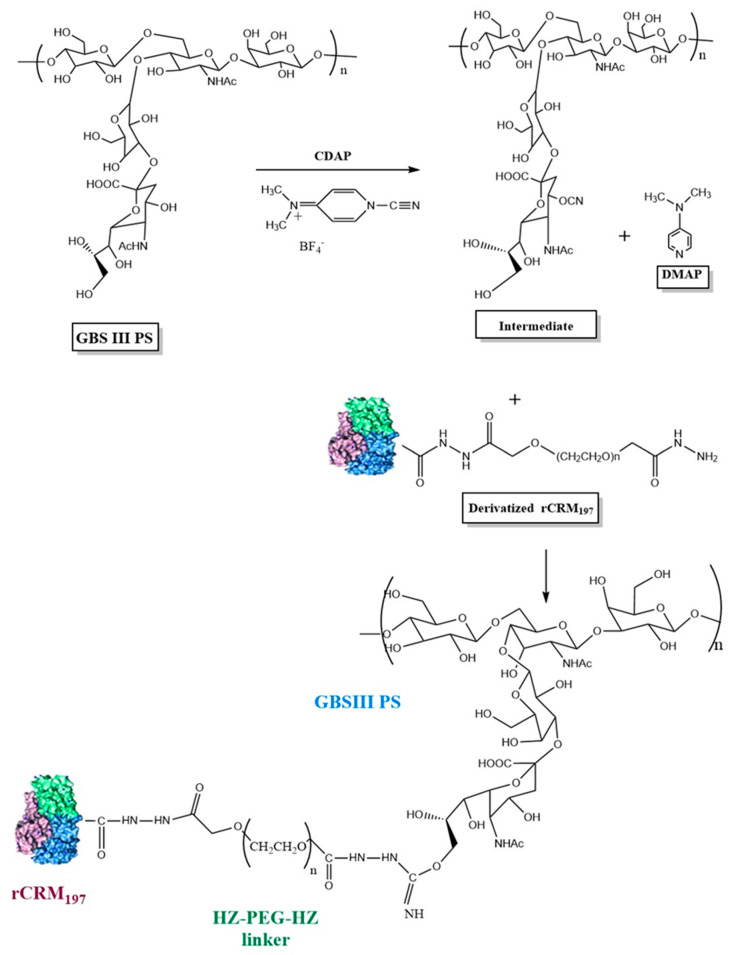
Schematic representation of GBS polysaccharide activation with CDAP and conjugation of activated GBS PS with derivatized rCRM197 using GBS III as a representative serotype. Structure of rCRM197 (shown as combination of green, blue, and purple colors) adapted from Gallagher et al. [31]. For illustration purposes, the C4 of sialic acid is shown as the PS activation site involving the -CN group, although other primary (C9 of sialic acid) and secondary hydroxyl groups of the constituent sugars are possible sites for PS activation and attachment to the derivatized rCRM_197_.

Purified GBS CPS was activated with 1-cyano-4-dimethylaminopyridinium tetrafluoroborate (CDAP) according to published methodology [28]. The conjugation scheme is provided in Figure 1. CDAP (SelectLab Chemicals, Münster, Germany) dissolved in acetonitrile (Millipore Sigma, St. Louis, MO, USA) was added to the polysaccharide at the desired PS:CDAP ratios and the pH was maintained at 9.0–10.0 with NaOH. After a 7–10 min activation period, rCRM197-linker was added to PS at 1:0.5–1:1.5 (w/w, PS: protein). After 3.0–6.0 h of conjugation, the reaction was quenched with 2 M Glycine (Thermo Fisher Scientific, Waltham, MA, USA). Conjugates were purified by tangential flow filtration (TFF) using a 300 kDa cut-off membrane cassette (Sartorius Stedim North America, Bohemia, NY, USA) in 20 mM L-histidine (Avantor Life Sciences, Radnor, PA, USA), 150 mM NaCl, pH 7.0 buffer and filtered through a 0.22 µm filter (Sartorius Stedim, Göttingen, Germany, Bohemia, NY, USA) and stored at 5 ± 3 °C.

Conjugates were characterized for PS and protein contents by Resorcinol [25] and Bicinchoninic acid assays (BCA) [29], respectively. Percentage of free PS in the conjugates was determined by deoxycholate-HCl precipitation followed by Resorcinol assay [30]. The molar mass of the conjugates was determined by the size exclusion chromatography multi-angle laser light scattering (SEC-MALLS) technique using a DAWN 8 system provided with a light scattering detector (Wyatt Technologies, Santa Barbara, CA, USA), and Arc Premier HPLC separation module, UV detector, and refractive index detector (Waters Corp., Milford, MA, USA).

### 2.4. GBS-06 Test Article Preparation

GBS-06 formulation comprising of monovalent bulk conjugates of ST Ia, Ib, II, III, V, and VII was prepared at a 20 µg dose for each of the six CPS antigens (120 µg total CPS) in 20 mM L-histidine buffer with 150 mM NaCl, pH 7.0 without adjuvant. The dose was selected to assess toxicity and immunogenicity at the highest foreseen clinical dose of 20 µg. The test article was confirmed to have very low endotoxin levels (<0.1 EU/µg of PS) and be bioburden-free before use in GLP toxicology studies.

### 2.5. GLP Toxicology Study of 6-Valent GBS Vaccine in Rabbits

New Zealand White (NZW) rabbits were chosen since preclinical immunogenicity has been well established in this species and they are the representative animal model of choice for the repeat dose toxicology study. Only animals that were in a good state of health as determined by a licensed veterinarian were included in the study. The animal sample size was determined based on the existing information and experience in performing GLP toxicology studies in animals. For animal inclusion and grouping, the animals were weighed, arranged in ascending order of their body weights, and weight-stratified rabbits were randomly distributed to all dose groups using a Microsoft Excel spreadsheet, such that the body weight variation of the animals did not exceed ± 20% of the mean body weight for each sex. Further, body weights of the animals were analyzed statistically for mean body weight to confirm that there was no statistically significant difference among groups within each sex. The body weight ranged from 1.80 to 2.20 kg across both the sexes and the animals were 13 weeks old at treatment. Each main group consisted of 6 rabbits/sex and each recovery (R) group consisted of 4 rabbits/sex. A three-dose regimen was selected based on an *n* + 1 schedule where “*n*” represents the possible number of doses in a clinical setting. In the unblinded study, a total of 20 rabbits (10 male and 10 female) received 0.9% saline (Groups G1/G1R) or unadjuvanted GBS-06 formulation at 20 μg of each of the six GBS serotypes/dose (Groups G2/G2R) via IM injection into the right thigh muscle at 0.5 mL/animal on Days 1, 22, and 43 of the experimental periods. After the last treatment on Day 43, the main group of animals (G1 and G2) were euthanized on Day 49; animals in the recovery groups (G1R and G2R) were euthanized on Day 71.

Study endpoints included mortality, clinical signs of toxicity, detailed clinical examinations, local injection site reactions, body weights, food consumption, ophthalmology examinations, body temperatures, clinical pathology parameters (hematology, coagulation, clinical chemistry, and urinalysis), C-reactive protein (CRP), immunogenicity, organ weights, and macroscopic and microscopic observations. The sera from the study were collected on the day prior to the first vaccination (pre-dose), prior to vaccination on Days 22 and 43, Day 49, and Day 71, and processed and stored at –80 °C.

### 2.6. Multiplex ELISA

Determination of anti-GBS capsular polysaccharide-specific immunoglobulin G responses (anti-GBS CPS-specific IgG responses) against each of the six serotypes (Ia, Ib, II, III, V, and VII) in the 6-valent formulation was carried out using a multiplexed bead-based immuno assay (MBIA, Bio-Rad, Hercules, CA, USA) [32]. The rabbit anti-GBS PS-specific serum IgG antibody concentrations for each of the six PS serotypes in the vaccine were tested by using an in-house qualified rabbit polyclonal standard reference serum with known concentrations of serotype-specific IgG antibody against each of the six serotypes (Ia, Ib, II, III, V, and VII). Single analysis was run with eight serial dilutions for each serum sample. IgG geometric mean concentrations (GMCs) were determined by calculation of eight determinations for each sample. The GMCs and 95% confidence interval (CI) were calculated for sera samples at all timepoints for each dosed group. Data analysis was performed using GraphPad Prism 10. Differences in serum IgG concentrations between groups were assessed using the Mann–Whitney U test, due to the non-normal distribution of the data and the small sample size. Prior to analysis, the distributions of IgG concentrations were confirmed to have similar shapes across groups, allowing for valid comparison using the Mann–Whitney U test. A significant *p*-value (*p* < 0.05 or *p* < 0.01 or *p* < 0.001) indicated that the geomean IgG concentration differences between the groups were statistically significant.

## 3. Results

### 3.1. Vaccine Characterization

**Figure 2:**
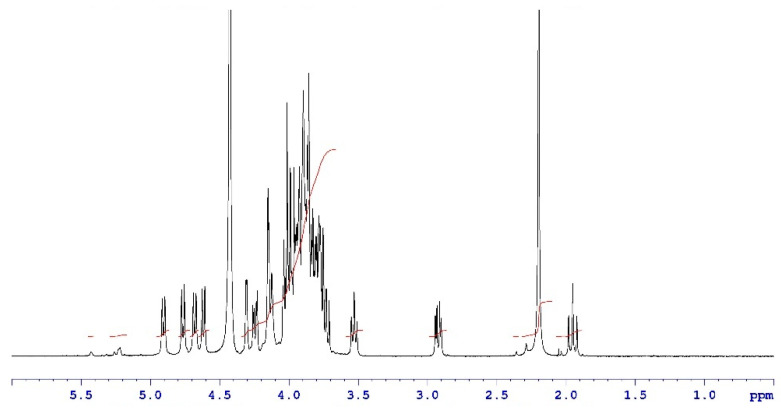
Structural identity of a representative GBS III PS by ^1^H-NMR. Data were acquired at a 400 MHz frequency at 75 °C and as an average of 40 scans. Chemical shifts are shown as black traces and the peak integrals are shown in red.

**Figure 3:**
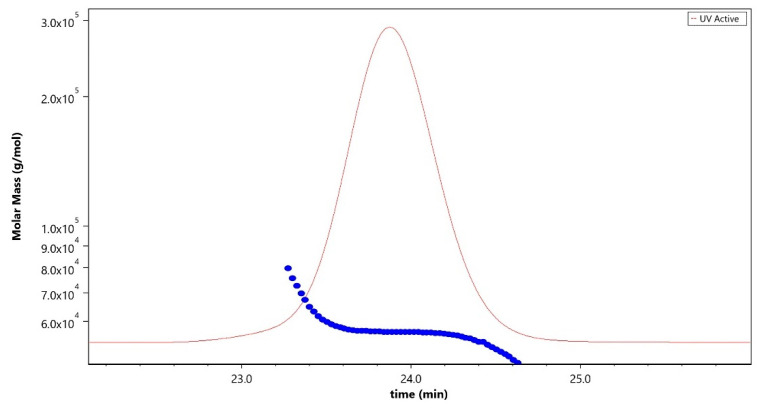
Molar mass profile (blue trace) of purified rCRM197 carrier protein analyzed using the SEC-MALLS technique corresponding to 58.1 kDa. The sample was run using LB-804 and LB-806 tandem columns at a flow rate of 0.8 mL/min with 1X PBS, pH 7.4 as the mobile phase buffer. The red trace denotes the UV signal at 280 nm.

Analytical characterization of the CPS, rCRM197 carrier protein, monovalent bulk conjugates, and the vaccine formulation was carried out using in-house established analytical methods. Appropriate in-process controls and release specifications were made available at each stage of vaccine development, and the product was ensured for quality as per internal quality guidelines prior to the initiation of the GLP toxicology study. The structural identity of the six capsular polysaccharides was determined by ^1^H-NMR and found to be consistent with published literature information [33]. A representative GBS III PS (Figure 2) shows the chemical shifts corresponding to the four anomeric protons from H_1_-β-GlcNAc (4.8 ppm), H_1_-β-Gal1-branch (4.65 ppm), H_1_-β-Glc (4.55 ppm) and H_1_-β-Gal2 (4.46 ppm), ring protons corresponding to H_6_-β-GlcNAc (4.21 ppm), H_4_-β-Gal2 (4.18 ppm), H_3_-β-Gal1-branch (4.1 ppm), H_2_-β-Glc (3.4 ppm), H_3eq_ NeuNAc (2.8 ppm), and H_3ax_ NeuNAc (1.8 ppm). Methyl protons corresponding to the N-acetyl groups of GlcNAc and NeuNAc (2.06 ppm) were also identified. ^1^H-NMR for PS Ia, Ib, II, V, and VII are provided as Figures S1–S5. The molar mass for rCRM197 carrier protein as determined by SEC-MALS was 58.1 kDa, which is consistent with the theoretical value of 58.4 kDa (Figure 3). The degree of derivatization of the rCRM197 protein with hydrazide linker was 2–3 based on TNBS assay. Product quality attributes of monovalent bulk conjugates such as conjugate molecular size, polysaccharide to rCRM197 carrier protein ratio, and % free PS (Table 1), antigenicity (Table S1), and identity (Table S1) were determined using in-house established analytical methods. The conjugates’ molecular size ranged from 2000 to 10,000 kDa (size exclusion profiles shown in Figures S6–S11) with a PS: protein of 0.7–1.1 (wt/wt). The free PS% was found to be low and <10% for each of the six monovalent bulk conjugates. The quality attributes were found to be optimal according to WHO Technical Review Series (TRS) guidelines available for other conjugate vaccines [34]. A test article containing the six monovalent conjugates formulated with excipients was tested in a repeat dose toxicology study in rabbits to evaluate safety and immunogenicity.

### 3.2. Vaccine Safety

There was no mortality and no GBS-06-related effects on clinical observations, body weights, percent body weight change with respect to Day 1, food consumption, ophthalmology findings, local injection site reactions, clinical pathology parameters (hematology, coagulation, clinical chemistry, and urinalysis), CRP, organ weights, or macroscopic or microscopic findings (manuscript in preparation).

### 3.3. Vaccine Immunogenicity

**Figure 4:**
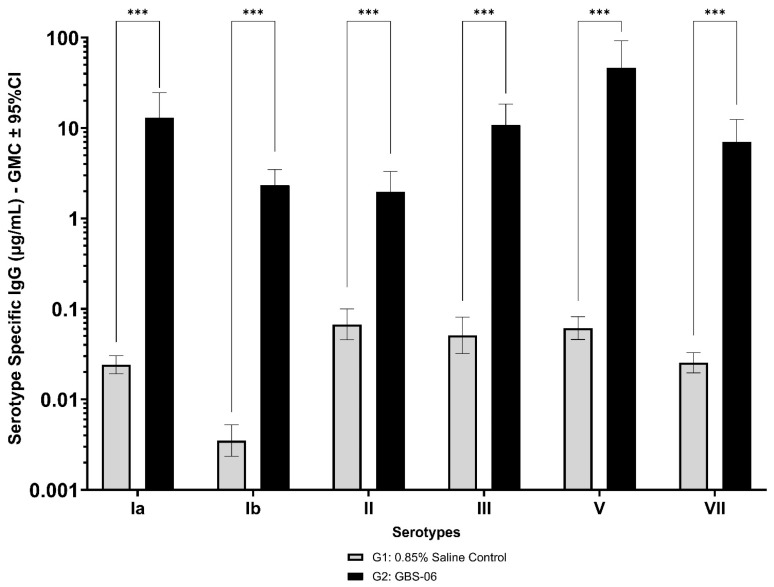
Comparison of anti-GBS PS serotype-specific IgG concentrations in Day 49 sera from NZW rabbits, 7 days following the third IM immunization with GBS-06 (G2) or 0.9% saline (G1). Data are represented as the geomean of 20 animals in each group. The geomean of eight determinations was calculated for each sample. Error bars represent 95% confidence intervals (CI) of the geomean for each group. The Mann–Whitney U test was applied for the assessment of differences in sera IgG antibody concentrations between groups. *** *p* < 0.001 indicates statistically significant differences in immune responses in rabbit sera following dosing with the control and GBS-06 test articles.

**Figure 5:**
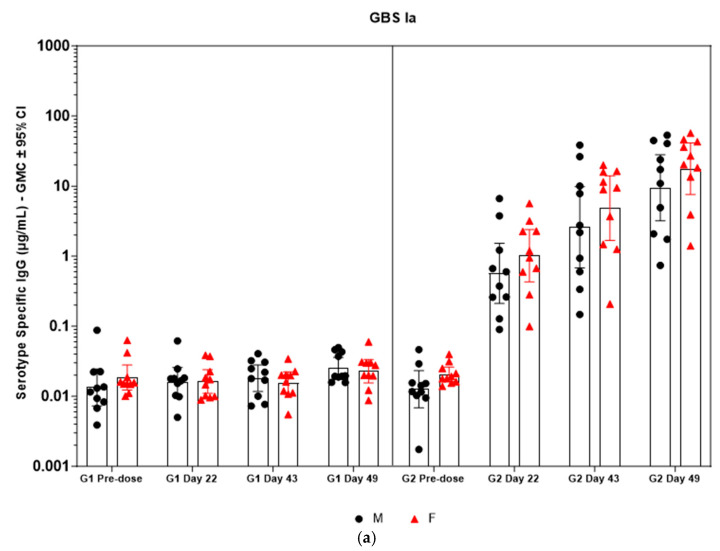

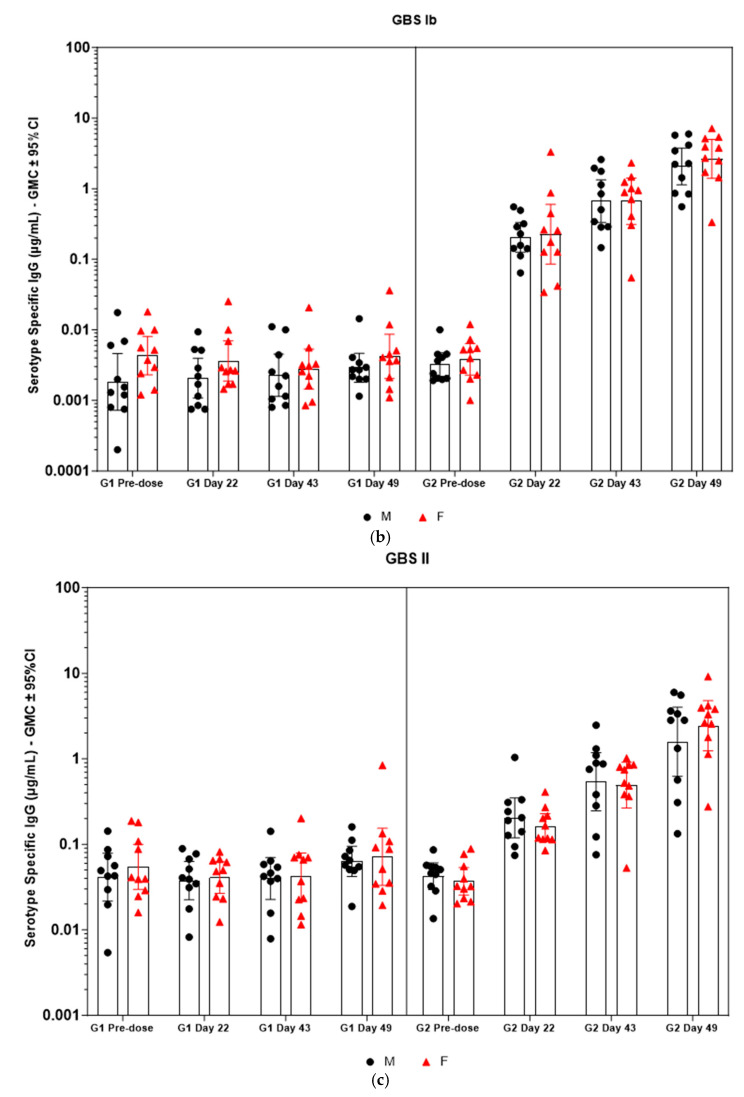

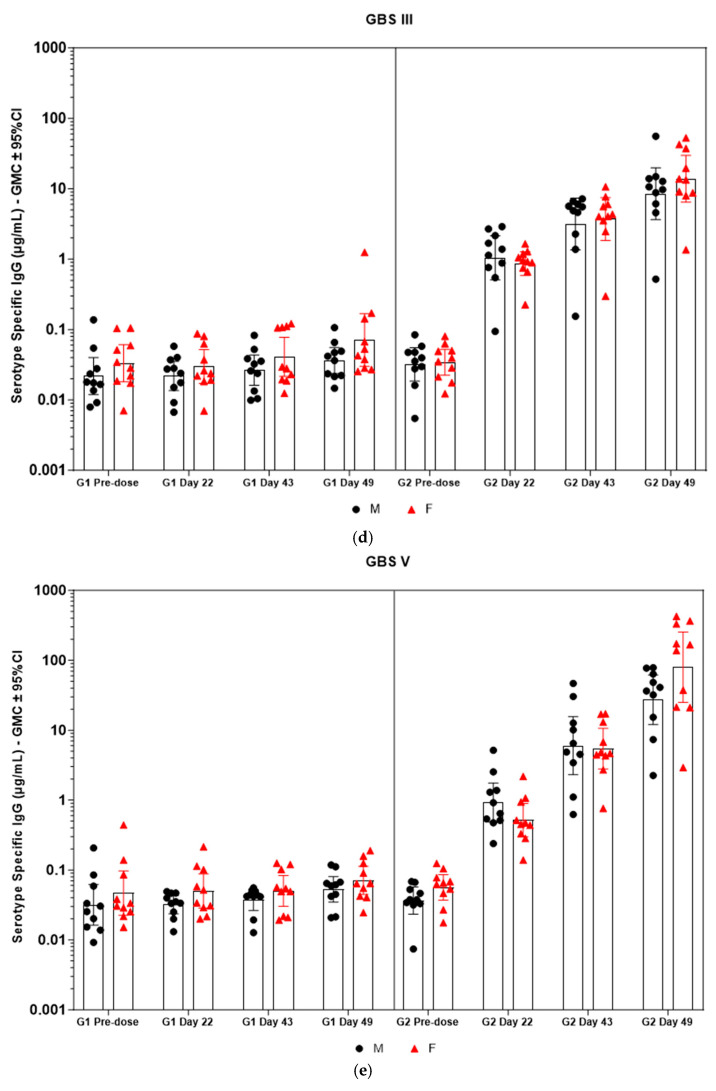

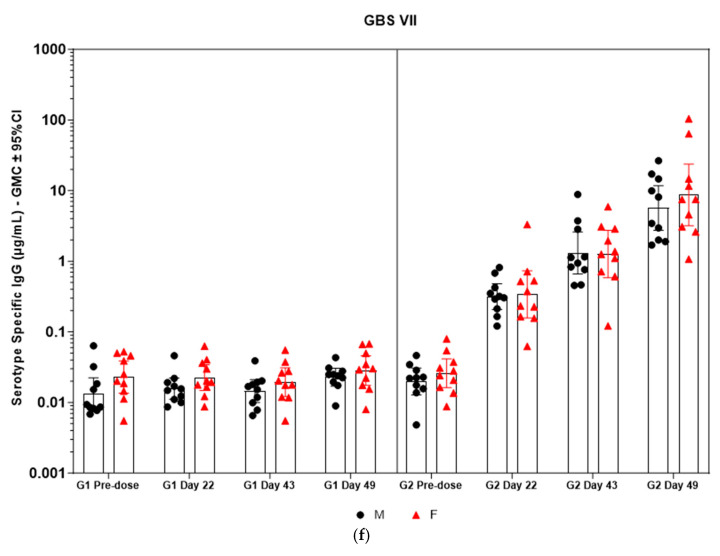
(**a**–**f**) Individual rabbit (grouped as male and female rabbits) anti-GBS PS-specific IgG antibody geometric mean concentrations against ST Ia, Ib, II, III, V, and VII and 95% confidence intervals in sera samples following one, two, and three intramuscular administrations of saline control (G1) and GBS test article (G2) (CI = confidence interval; M = male; and F = female). Single analysis was run with eight serial dilutions for each serum sample. IgG geomean concentrations were determined by calculation of eight determinations for each sample. The Mann–Whitney U test was applied for comparison of differences in antibody concentrations between male and female rabbits in the groups and no statistically significant differences in immune responses was observed between the two sexes (*p* > 0.05).

Immunogenicity data from sera corresponding to the main group of rabbits (pre-dose, Day 22, Day 43, and Day 49) from control and test formulations are reported in this study. In the saline control group (G1), baseline anti-GBS PS-specific IgG antibody GMCs from pre-dose sera ranged from 0.003 to 0.038 µg/mL for the six serotypes. Following the dosing on Day 1, Day 22, and Day 43, there were no changes in antibody responses for all the six serotypes, and IgG GMCs at Day 49 was 0.024 µg/mL for Ia, 0.004 µg/mL for Ib, 0.067 µg/mL for II, 0.051 µg/mL for III, 0.061 µg/mL for V, and 0.025 µg/mL for VII (Table 2). The results are as expected for naïve rabbits following immunization with the control article. In the GBS-06 group (G2), very low immune responses were detected in the pre-dose sera with the anti-GBS PS-specific IgG antibody GMCs of 0.016 µg/mL, 0.003 µg/mL, 0.040 µg/mL, 0.033 µg/mL, 0.045 µg/mL, and 0.023 µg/mL for Ia, Ib, II, III, V, and VII, respectively (Table 3). Thus, the baseline IgG GMCs from the GBS-06 pre-dose sera was similar to that of the control sera. Following the first dose with the GBS-06 article, there was an increase in sera anti-GBS PS-specific IgG responses for each of the six PS serotypes on Day 22 with the GMCs of 0.760 µg/mL, 0.215 µg/mL, 0.182 µg/mL, 0.952 µg/mL, 0.689 µg/mL, and 0.327 µg/mL for Ia, Ib, II, III, V, and VII, respectively (Table 3). The same trend of boost in immune responses was observed in Day 43 sera after the second dose vaccination in all the six serotypes with the IgG GMCs of 3.540 µg/mL for Ia, 0.668 µg/mL for Ib, 0.515 µg/mL for II, 3.431 µg/mL for III, 5.715 µg/mL for V, and 1.287 µg/mL for VII (Table 3). The highest antibody concentrations were noted on Day 49 after the third vaccination with at least a 3-fold increase in IgG GMCs across all serotypes compared to that of responses after the second dose vaccination in Day 43 sera (Table 3). The greatest increases in anti-GBS PS-specific IgG GMCs in the test group were found to be 12.947 µg/mL at Day 49 vs. 0.016 µg/mL at pre-dose for GBS Ia; 2.335 µg/mL at Day 49 vs. 0.003 µg/mL at pre-dose for GBS Ib; 1.962 µg/mL at Day 49 vs. 0.040 µg/mL at pre-dose for GBS II; 10.873 µg/mL at Day 49 vs. 0.033 µg/mL at pre-dose for GBS III; 46.536 µg/mL at Day 49 vs. 0.045 µg/mL pre-dose for GBS V; and 7.028 µg/mL at Day 49 vs. 0.023 µg/mL at pre-dose for GBS VII. It should also be highlighted that the highest level of antibody response after the third dose (Day 49) was achieved for ST V (46.536 µg/mL) among the six serotypes. In addition, there were no significant differences in the IgG antibody responses observed in male and female rabbits across all the six serotypes in pre-dose, Day 22, Day 43, and Day 49 sera (*p* > 0.05). The anti-GBS PS-specific IgG GMCs in Day 49 sera from rabbits immunized with GBS-06 was significantly higher (*p* < 0.001) as compared to anti-GBS PS-specific IgG GMCs in rabbits immunized with the 0.9% saline control (Figure 4). The fold rise in IgG antibody responses in GBS-06 sera at the Day 22, Day 43, and Day 49 timepoints compared to that of the control sera are provided in Table 4. Individual rabbit (grouped as male and female rabbits) IgG GMCs in pre-dose, D22, D43, and D49 sera for each of the six ST-Ia, Ib, II, III, V, and VII in saline control or GBS-06 groups are provided in Figure 5a–f. Overall, strong immune responses were generated against each of the STs in both the male and female rabbits following dosing with the GBS-06 test article.

Table 2 shows the geometric mean concentrations and 95% confidence intervals in sera samples following one, two, or three intramuscular administrations of saline control (Group 1) (CI = confidence interval). Single analysis was run with eight serial dilutions for each serum sample. IgG geomean concentration was determined by calculation of eight determinations for each sample. LLOQ and ULOQ ranged between 0.035–0.130 ng/mL and 4.45–16.68 ng/mL, respectively, depending on the serotype.

Table 3 geometric mean concentrations and 95% confidence intervals in sera samples following one, two, or three intramuscular administrations of the GBS-06 test article (Group 2) (CI = confidence interval). Single analysis was run with eight serial dilutions for each serum sample. IgG geomean concentration was determined by calculation of eight determinations for each sample. LLOQ and ULOQ ranged between 0.035–0.130 ng/mL and 4.45–16.68 ng/mL, respectively, depending on the serotype. The samples were diluted in order to fit the working range.

Table 4 shows the fold rise in IgG geometric mean concentrations in Day 22, Day 43, and Day 49 sera samples following intramuscular administrations of GBS-06 (G2) in comparison to that of saline control (G1) at the respective timepoints. The greatest and the lowest fold IgG increase were observed for STs V and II, respectively, in the G2 group in comparison to the G1 group. IgG geomean concentration was determined by calculation of eight determinations for each sample.

## 4. Discussion

GBS disease caused by *Streptococcus agalactiae* species inflicts mortality and debilitating effects on millions of newborns. Recent data suggest that the global burden of GBS is higher than previously recognized. This includes GBS neonatal/early infant meningitis, sepsis, death, and neurodevelopmental impairment, with additional stillbirth/preterm births and maternal sepsis, with the burden being highest in sub-Saharan Africa [35]. Sero-epidemiological data suggest that certain concentrations of GBS serotype-specific maternal serum antibodies transferred in utero to infants are associated with a diminished risk of neonatal GBS sepsis [15,36]. Momentum for a more aggressive GBS maternal immunization effort has been building recently due to (1) the success of the maternal and neonatal tetanus (MNT) elimination initiative, (2) data demonstrating that vaccination of pregnant women with inactivated influenza vaccines, pertussis, and RSV vaccines confers protection against these infections in newborn infants that is persistent for the first few months of life [37], and (3) positive phase 1 and 2 clinical trial results from other GBS conjugate vaccines in non-pregnant/pregnant women [17,18], including the most recent clinical data from immunization of a Tdap-GBS combination vaccine in non-pregnant women volunteers [38]. In this study, we presented the development of a 6-valent conjugate vaccine, GBS-06, with a focus on the vaccine ST coverage, preclinical immunogenicity data, and advancements to the vaccine program. Significant findings from the GLP toxicology study reveal that GBS-06 is tolerated at the maximum evaluated dose of 20 µg in rabbits without any clinical signs of toxicity, or vaccine-related adverse effects combined with the induction of strong humoral responses against each of the six capsular polysaccharides antigens—Ia, Ib, II, III, V, and VII—in the test article in comparison with the placebo control group.

Development of a maternal GBS vaccine providing the broadest coverage involves an understanding of the serotype distribution from representative samples such as maternal rectovaginal tract, umbilical cord blood, and infant blood isolates, and different clinical presentations such as maternal colonization, maternal disease including preterm or stillbirths, and infant EOD and LOD. A comprehensive analysis of global GBS serotype distribution based on clinical isolates from maternal colonization and newborns indicates ST Ia, III, and V to be the predominant STs associated with neonatal disease. Collectively, a vaccine containing Ia, Ib, II, III, and V in its composition could prevent different manifestations of the disease by >90% [39]. However, emphasis on other emerging serotypes in the pregnant women population in LMICs settings or regions where IAP policies are either unavailable or not fully defined, is warranted. In this context, relevance and significance of the inclusion of ST VII as discussed in the current study should be highlighted. ST VII is one of the circulating serotypes in the maternal population in different parts of Eastern and Southeastern Asia and is associated with maternal and infant morbidities [9,19,20]. The juxtaposition of these findings with the ST composition in vaccines that are more advanced in clinical trials indicates that the vaccine coverage could be reduced in regions with the prevalence of ST VII [19]. Taken together, a 6-valent formulation containing Ia, Ib, II, III, V, and VII can provide protection against 99% of GBS serotypes causing stillbirth, 98% of serotypes causing EOD, and 99% of serotypes causing LOD [3], and can effectively address the issue of serotype replacement from the non-vaccine serotypes that are commonly observed with glycoconjugate vaccines.

We explored cyanylation chemistry involving CDAP coupled with the proprietary HZ-PEG-HZ linker platform technology for GBS-06 glycoconjugate development. The CDAP-based conjugation approach is widely known in the glycoconjugate vaccine field, and it has been adopted in approved pneumococcal and meningococcal vaccines [40,41]. With cyanylation being the conjugation chemistry of choice, we also employed HZ-PEG-HZ linker for derivatization of the rCRM197 carrier protein and subsequent conjugation to CPS. A review of the literature suggests that the inclusion of the PEG linker in meningococcal vaccines showed an enhancement of immune responses likely by preventing steric hindrance between carrier protein and a complex polymer such as polysaccharides [42]. The presence of PEG has also been shown to improve the half-life of antibody–drug conjugates and other PEG-based biopharmaceuticals by enabling slower serum clearance, presumably by delaying uptake by the macrophages and other immune effector cells [43]. Consistent with this idea, GBS-06 glycoconjugate vaccine development involving an HZ-PEG-HZ linker was carried out, evaluated in proof-of-concept preclinical studies, and found to generate strong immune responses without any safety concerns in animals. With promising immunogenicity data from several preclinical studies, a GLP toxicology study was conducted for comprehensive safety and immunogenicity assessment of the vaccine candidate before progression into clinical trials. Characterization of the CPS and rCRM197 starting materials used in the GBS-06 test article preparation for the toxicology study indicates that the structural identity of all six CPSs as determined by ^1^H-NMR was consistent with the published literature data [33]. Further, the presence of characteristic spectral shifts related to the axial and equatorial protons in sialic acid and methyl protons arising from the N-acetyl groups of the constituent amino sugars is confirmed since these structural features are key to vaccine immunogenicity [44]. Similarly, analytical characterization of rCRM197 by chromatography confirm protein purity with >90% protein monomer, thus ensuring the quality of the carrier protein that directly mediates T-cell responses to glycoconjugate vaccines and confers B cell memory (primarily through IgG antibodies) [45]. Characterization of the monovalent bulk conjugates involving key product attributes such as conjugate molar mass, PS: protein, and % free PS is optimal based on the guidelines available for other conjugate vaccines, although there is no WHO TRS currently available for production and control of GBS conjugate vaccines [34]. Of note, the free PS removal process is very efficient (<10% free PS) during the manufacturing of six conjugate drug substances. Higher free PS levels in the product can result in the vaccine being weakly immunogenic [46].

Immunogenicity analysis of sera from the GLP toxicology study of the GBS-06 vaccine shows negligible IgG antibody GMCs for the six serotypes in the saline control group (G1), as expected for naïve rabbits following immunization with the control article (Table 2). In the GBS-06 group (G2), there is an observable increase in serum anti-GBS PS-specific IgG antibody GMCs for each of the six PS serotypes on Day 22 after the first vaccination and Day 43 after the second vaccination, with the highest antibody concentrations noted on Day 49 after the third vaccination (Table 3). There is a >20-fold increase in sera IgG concentrations against each of the six serotypes in both the male and female rabbits following dosing with the GBS-06 article compared to that of the saline control group (*p* < 0.001), which is indicative of the elicitation of strong immune responses by the 6-valent GBS glycoconjugate vaccine (Figure 4) and (Figure 5). A limitation of the current study is the lack of functional antibody response data against the GBS-06 vaccine candidate containing a 20 µg antigen dose. Nevertheless, functional antibody analysis from previous representative batches of GBS-06 was performed using opsonophagocytic killing assay (OPA) at different antigen doses (5 and 10 µg). Higher bacterial killing titers (> 800) were achieved against each of the circulating bacterial strains corresponding to the six STs included in GBS-06 with strong correlations to the neutralizing antibody responses at these doses compared to the placebo control (killing titers < 8) (Table S2). Taken together, our observations are highly suggestive of the effectiveness of the GBS-06 vaccine in eliciting protective antibody responses against GBS disease.

Establishing correlates of protection is critical for demonstration of vaccine efficacy and providing a regulatory pathway for licensure. One of the approaches that is under discussion for determination of GBS vaccine efficacy is based on the serological thresholds of risk reduction (SToRR) endpoints. For example, measuring binding and/or functional antibody levels in cord blood at infant birth can serve as an SToRR [47]. In a sero-epidemiological study in South Africa conducted as a subset of the GBS-6 glycoconjugate vaccine clinical trial in pregnant women [NCT01755598], anti-CPS pooled IgG concentration thresholds of 0.184 µg/mL were predicted to provide a 75% reduction in GBS disease risk based on Bayesian statistics [17]. In another study involving the phase I clinical trial of the GBS subunit vaccine in healthy adult women, IgG thresholds of ≥0.428 µg/mL and ≥0.112 µg/mL against RibN and Alp1N cell-surface proteins, respectively, were associated with a 90% reduction in invasive GBS disease based on a case-control natural immunity study performed again in South Africa [48]. Hence, SToRR-based clinical trials combined with post-licensure vaccine effectiveness studies can accelerate approval and enable timely availability of vaccines, especially in LMIC settings. A similar approach could be potentially applied as a regulatory strategy in GBS-06 clinical trials for vaccine licensure. Albeit there is a lack of clinical data on GBS-06 vaccine efficacy, the serology data in rabbits from the current study indicate induction of robust immune responses against the six capsular polysaccharide antigens compared to the placebo, with the caveat that the differences in dosing schedule and the nature of immune responses in preclinical models cannot faithfully predict the vaccine efficacy in humans.

Success of GBS vaccines for maternal immunization involves careful product development considerations given the target population being pregnant women. The rCRM197 carrier protein, which is a component in the GBS-06 glycoconjugate vaccine, is known for its precedence of use in licensed pediatric vaccines against *S. pneumoniae* and *N. meningitidis* [49]. A positive safety profile for PEG has been established based on approved COVID vaccines, of which millions of doses have been administered in the pregnant women population [50]. As for the effects of hydrazide linker on patient safety, hydrazine, a congener of hydrazide, has previously been used in the meningococcal conjugate vaccine MenAfriVac in pregnant and lactating women, in addition to the target pediatric population, without any safety concerns [51]. In addition, a positive safety profile for the hydrazide PEG linker has been established based on phase I clinical trial data of a pneumococcal conjugate vaccine candidate employing the same linker technology from our group in healthy non-pregnant adults (NCT05540028), although direct evaluation of the 6-valent GBS vaccine in pregnant women is warranted. In regard to the vaccine dose selection and composition, earlier non-clinical immunogenicity work involved antigen dose-ranging studies and assessment of adjuvant effects in augmenting CPS antigen-based IgG immune responses. The data from the previous studies indicate induction of robust IgG immune responses across the antigen doses tested (2.5–20 µg), which was further enhanced by the presence of adjuvant in the test articles compared to that of non-adjuvanted formulation however, adjuvanted formulation was not included in later studies because the enhancement of immune response is not anticipated in adult humans. Data from a phase 1/2 study in healthy adults (men and nonpregnant women) with a similar rCRM_197_-conjugated 6-valent GBS conjugate vaccine candidate formulated with and without aluminum phosphate revealed that the vaccine elicited robust immune responses that persisted for 6 months after vaccination irrespective of the presence of adjuvant [52]. Owing to the lack of an immunological benefit of including an adjuvant in the vaccine formulation and consistent with the WHO preferred product characteristics for a GBS vaccine without adjuvant [53], a non-adjuvanted formulation at the highest clinical dose of 20 µg has been evaluated in the GLP toxicology study.

## 5. Conclusions

To our knowledge, this is the first report of a HZ-PEG-HZ linker-based GBS glycoconjugate vaccine to be evaluated in a GLP toxicology study. Additionally noteworthy is the unique vaccine composition of GBS-06-containing ST VII. Analytical characterization data of the six monovalent conjugates reveal optimal product quality attributes, which is further confirmed by induction of significant and robust anti-GBS capsular PS-specific IgG antibody responses against all six GBS serotypes in both male and female rabbits. The results of the immunogenicity analysis show that GBS-06 is pharmacologically active. The candidate is currently being evaluated at three dose levels: 2.5 µg, 7.5 µg, and 20.0 µg in phase I clinical trials in healthy, non-pregnant women volunteers in the U.S and South Africa (NCT06611371). Positive results from the clinical trials would signal a step closer to the approval and licensure of a glycoconjugate vaccine for effective management of GBS disease affecting millions of newborns worldwide.

## Figures and Tables

**Table 1:** Analytical characterization data for the derivatized rCRM carrier protein and six monovalent bulk conjugates used in the preparation of GBS-06 vaccine formulation.

Serotype	PS: Protein (wt/wt)	% Free PS	Molar Mass (M_w_*, kDa)
Ia	1.1	4	7844
Ib	0.8	5	7364
II	0.7	3	4532
III	0.7	3	7433
V	0.9	4	2443
VII	0.7	3	9531

M_w_* denotes the weight-averaged molar mass distribution of molecules determined by the SEC-MALLS technique.

**Table 2:** Anti-GBS serotype-specific IgG concentrations in sera following one, two, or three intramuscular administrations of saline control (G1) in NZW rabbits.

Group 1	GBS Ia	GBS Ib	GBS II	GBS III	GBS V	GBS VII
Number of animals in per-protocol population	*n* = 20 (10/sex)					
Number of animals with evaluable anti-GBS PS-specific IgG concentrations	*n* = 20	*n* = 20	*n* = 20	*n* = 20	*n* = 20	*n* = 20
Pre-dose GMC (95% CI)	0.016 (0.011, 0.022)	0.003 (0.002, 0.005)	0.047 (0.032, 0.071)	0.027 (0.018, 0.040)	0.038 (0.024, 0.061)	0.017 (0.012, 0.025)
Day 22 GMC (95% CI)	0.016 (0.012, 0.021)	0.003 (0.002, 0.004)	0.039 (0.029, 0.053)	0.026 (0.018, 0.036)	0.040 (0.029, 0.054)	0.019 (0.014, 0.024)
Day 43 GMC (95% CI)	0.017 (0.013, 0.022)	0.003 (0.002, 0.004)	0.041 (0.028, 0.060)	0.033 (0.023, 0.048)	0.043 (0.032, 0.057)	0.017 (0.013, 0.022)
Day 49 GMC (95% CI)	0.024 (0.019, 0.030)	0.004 (0.002, 0.005)	0.067 (0.045, 0.100)	0.051 (0.032, 0.081)	0.061 (0.046, 0.082)	0.025 (0.020, 0.033)

**Table 3:** Anti-GBS serotype-specific IgG geomean concentrations in sera following one, two, or three intramuscular administrations of GBS-06 (G2) in NZW rabbits.

Group 2	GBS Ia	GBS Ib	GBS II	GBS III	GBS V	GBS VII
Number of animals in per-protocol population	*n* = 20 (10/sex)					
Number of animals with evaluable anti-GBS PS-specific IgG concentrations	*n* = 20	*n* = 20	*n* = 20	*n* = 20	*n* = 20	*n* = 20
Pre-dose GMC (95% CI)	0.016 (0.012, 0.022)	0.003 (0.003, 0.005)	0.040 (0.031, 0.050)	0.033 (0.024, 0.045)	0.045 (0.034, 0.061)	0.023 (0.017, 0.030)
Day 22 GMC (95% CI)	0.760 (0.414, 1.395)	0.215 (0.132, 0.352)	0.182 (0.136, 0.244)	0.952 (0.657, 1.379)	0.689 (0.459, 1.033)	0.327 (0.221, 0.486)
Day 43 GMC (95% CI)	3.540 (1.620, 7.735)	0.668 (0.420, 1.061)	0.515 (0.328, 0.807)	3.431 (2.090, 5.630)	5.715 (3.376, 9.677)	1.287 (0.810, 2.045)
Day 49 GMC (95% CI)	12.947 (6.838, 24.515)	2.335 (1.569, 3.474)	1.962 (1.158, 3.325)	10.873 (6.419, 18.417)	46.536 (23.374, 92.647)	7.028 (3.977, 12.420)

**Table 4:** Fold rise in anti-GBS serotype-specific IgG GMCs at different timepoints following intramuscular administrations of GBS-06 (G2) in NZW rabbits compared to that of saline control group (G1).

	GBS Ia	GBS Ib	GBS II	GBS III	GBS V	GBS VII
Fold rise in anti-GBS PS-specific IgG GMCs in GBS-06 compared to placebo Day 22	47.5	71.7	4.7	36.6	17.2	17.2
Fold rise in anti-GBS PS-specific IgG GMCs in GBS-06 compared to placebo Day 43	208.2	222.7	12.6	103.9	132.9	75.7
Fold rise in anti-GBS PS-specific IgG GMCs in GBS-06 compared to placebo Day 49	539.5	583.8	29.3	213.2	762.9	281.1

## Data Availability

The data presented in this study is available in this article.

## Supplementary Materials

The following supporting information can be downloaded at: https://www.mdpi.com/article/10.3390/vaccines13090952/s1, Figure S1: Proton NMR of GBS Ia PS, Figure S2: Proton NMR of GBS Ib PS, Figure S3: Proton NMR of GBS II PS, Figure S4: Proton NMR of GBS V PS, Figure S5: Proton NMR of GBS VII PS, Figure S6: Molar mass exclusion profile of GBS Ia conjugate, Figure S7: Molar mass exclusion profile of GBS Ib conjugate, Figure S8: Molar mass exclusion profile of GBS II conjugate, Figure S9: Molar mass exclusion profile of GBS III conjugate, Figure S10: Molar mass exclusion profile of GBS V conjugate, Figure S11: Molar mass exclusion profile of GBS VII conjugate; Table S1: Antigenicity, antigen content, and identity of each of the six conjugated GBS capsular polysaccharides in the 6-valent GBS conjugate vaccine (GBS-06) used in GLP Toxicology Study, Table S2: Functional antibody responses for a representative batch of GBS-06 vaccine using OPA.

## Abbreviations

The following abbreviations are used in this manuscript:

BCA	Bicinchoninic Acid Assay
CDAP	1-cyano-4-dimethylaminopyridinium tetrafluoroborate
CPS	Capsular polysaccharide
EDC	1-Ethyl-3-(3-dimethylaminopropyl)carbodiimide)
EOD	Early Onset Disease
GBS	Group B Streptococcus
GMC(s)	Geometric Mean concentration(s)
HZ-PEG-HZ	Hydrazide-Polyethylene Glycol-Hydrazide
IAP	Intrapartum Antibiotic Prophylaxis
IgG	Immunoglobulin G
LOD	Late Onset Disease
LMICs	Low- and Middle-Income Countries
MBIA	Multiplexed Bead-based Immunoassay
MNT	Maternal and Neonatal Tetanus
NZW	New Zealand White
OPA	Opsonophagocytic Killing Assay
PS	Polysaccharide
rCRM197	recombinant Cross-Reacting Material_197_
SEC-MALLS	Size Exclusion Chromatography Multi Angle Laser Light Scattering
ST	Serotype
SToRR	Serological Thresholds of Risk Reduction
TMP	Trans Membrane Pressure
TNBS	2,4,6-trinitrobenzene sulfonic acid

## References

[1] Patras K.A., Nizet V. (2018). Group B Streptococcal Maternal Colonization and Neonatal Disease: Molecular Mechanisms and Preventative Approaches. Front. Pediatr..

[2] Madrid L., Seale A.C., Kohli-Lynch M., Edmond K.M., Lawn J.E., Heath P.T., Madhi S.A., Baker C.J., Bartlett L., Cutland C. (2017). Infant Group B Streptococcal Disease Incidence and Serotypes Worldwide: Systematic Review and Meta-Analyses. Clin. Infect. Dis..

[3] Seale A.C., Bianchi-Jassir F., Russell N.J., Kohli-Lynch M., Tann C.J., Hall J., Madrid L., Blencowe H., Cousens S., Baker C.J. (2017). Estimates of the Burden of Group B Streptococcal Disease Worldwide for Pregnant Women, Stillbirths, and Children. Clin. Infect. Dis..

[4] Kolter J., Henneke P. (2017). Codevelopment of Microbiota and Innate Immunity and the Risk for Group B Streptococcal Disease. Front. Immunol..

[5] Carl M.A., Ndao I.M., Springman A.C., Manning S.D., Johnson J.R., Johnston B.D., Burnham C.-A.D., Weinstock E.S., Weinstock G.M., Wylie T.N. (2014). Sepsis from the Gut: The Enteric Habitat of Bacteria That Cause Late-Onset Neonatal Bloodstream Infections. Clin. Infect. Dis..

[6] Melin P. (2011). Neonatal Group B Streptococcal Disease: From Pathogenesis to Preventive Strategies. Clin. Microbiol. Infect..

[7] Davies H.G., Carreras-Abad C., Le Doare K., Heath P.T. (2019). Group B Streptococcus: Trials and Tribulations. Pediatr. Infect. Dis. J..

[8] WHO (2021). Group B Streptococcus Vaccine: Full Value of Vaccine Assessment. Executive Summary.

[9] Kwatra G., Izu A., Cutland C., Akaba G., Ali M.M., Ahmed Z., Beck M.M., Barsosio H.C., Berkley J.A., Chaka T.E. (2024). Prevalence of Group B Streptococcus Colonisation in Mother-Newborn Dyads in Low-Income and Middle-Income South Asian and African Countries: A Prospective, Observational Study. Lancet Microbe.

[10] Alotaibi N.M., Alroqi S., Alharbi A., Almutiri B., Alshehry M., Almutairi R., Alotaibi N., Althoubiti A., Alanezi A., Alatawi N. (2023). Clinical Characteristics and Treatment Strategies for Group B Streptococcus (GBS) Infection in Pediatrics: A Systematic Review. Medicina.

[11] Carreras-Abad C., Ramkhelawon L., Heath P.T., Le Doare K. (2020). A Vaccine Against Group B Streptococcus: Recent Advances. IDR.

[12] Paoletti L.C., Kasper D.L. (2019). Surface Structures of Group B *Streptococcus* Important in Human Immunity. Microbiol. Spectr..

[13] Jennings H.J., Rosell K.G., Kasper D.L. (1980). Structural Determination and Serology of the Native Polysaccharide Antigen of Type-III Group B Streptococcus. Can. J. Biochem..

[14] Baker C.J., Kasper D.L. (1976). Correlation of Maternal Antibody Deficiency with Susceptibility to Neonatal Group B Streptococcal Infection. N. Engl. J. Med..

[15] Baker C.J., Kasper D.L. (1985). Group B Streptococcal Vaccines. Rev. Infect. Dis..

[16] Bjerkhaug A.U., Ramalingham S., Mboizi R., Le Doare K., Klingenberg C. (2024). The Immunogenicity and Safety of Group B Streptococcal Maternal Vaccines: A Systematic Review. Vaccine.

[17] Madhi S.A., Anderson A.S., Absalon J., Radley D., Simon R., Jongihlati B., Strehlau R., Van Niekerk A.M., Izu A., Naidoo N. (2023). Potential for Maternally Administered Vaccine for Infant Group B Streptococcus. N. Engl. J. Med..

[18] Gonzalez-Miro M., Pawlowski A., Lehtonen J., Cao D., Larsson S., Darsley M., Kitson G., Fischer P.B., Johansson-Lindbom B. (2023). Safety and Immunogenicity of the Group B Streptococcus Vaccine AlpN in a Placebo-Controlled Double-Blind Phase 1 Trial. iScience.

[19] Islam M.S., Saha S.K., Islam M., Modak J.K., Shah R., Talukder R.R., El Arifeen S., Baqui A.H., Darmstadt G.L., Mullany L.C. (2016). Prevalence, Serotype Distribution and Mortality Risk Associated With Group B Streptococcus Colonization of Newborns in Rural Bangladesh. Pediatr. Infect. Dis. J..

[20] Ghia C., Rambhad G. (2021). Disease Burden Due to Group B *Streptococcus* in the Indian Population and the Need for a Vaccine–a Narrative Review. Ther. Adv. Infect..

[21] Russell N.J., Seale A.C., O’Driscoll M., O’Sullivan C., Bianchi-Jassir F., Gonzalez-Guarin J., Lawn J.E., Baker C.J., Bartlett L., Cutland C. (2017). Maternal Colonization with Group B Streptococcus and Serotype Distribution Worldwide: Systematic Review and Meta-Analyses. Clin. Infect. Dis..

[22] Creti R., Imperi M., Gherardi G., Alfarone G., Marani I., Vocale C., Berardi A., Truocchio S., Miselli F. (2025). Group B Streptococcus (GBS) Carriage in Pregnant Women: Possible Emergence of Rare Serotypes and Antibiotic Resistance in Neonatal Disease. Microorganisms.

[23] Datta A., Kapre K., Andi-Lolo I., Kapre S. (2022). Multi-Valent Pneumococcal Conjugate Vaccine for Global Health: From Problem to Platform to Production. Hum. Vaccines Immunother..

[24] Padín-González E., Lancaster P., Bottini M., Gasco P., Tran L., Fadeel B., Wilkins T., Monopoli M.P. (2022). Understanding the Role and Impact of Poly (Ethylene Glycol) (PEG) on Nanoparticle Formulation: Implications for COVID-19 Vaccines. Front. Bioeng. Biotechnol..

[25] Svennerholm L. (1957). Quantitive Estimation of Sialic Acids: II. A Colorimetric Resorcinol-Hydrochloric Acid Method. Biochim. Biophys. Acta.

[26] Kossaczka Z., Bystricky S., Bryla D.A., Shiloach J., Robbins J.B., Szu S.C. (1997). Synthesis and Immunological Properties of Vi and Di-O-Acetyl Pectin Protein Conjugates with Adipic Acid Dihydrazide as the Linker. Infect. Immun..

[27] Okuyama T., Satake K. (1960). On The Preparation and Properties of 2, 4, 6-Trinitrophenyl-Amino Acids and-Peptides. J. Biochem..

[28] Shafer D.E., Toll B., Schuman R.F., Nelson B.L., Mond J.J., Lees A. (2000). Activation of Soluble Polysaccharides with 1-Cyano-4-Dimethylaminopyridinium Tetrafluoroborate (CDAP) for Use in Protein-Polysaccharide Conjugate Vaccines and Immunological Reagents. II. Selective Crosslinking of Proteins to CDAP-Activated Polysaccharides. Vaccine.

[29] Smith P.K., Krohn R.I., Hermanson G.T., Mallia A.K., Gartner F.H., Provenzano M.D., Fujimoto E.K., Goeke N.M., Olson B.J., Klenk D.C. (1985). Measurement of Protein Using Bicinchoninic Acid. Anal. Biochem..

[30] Lei Q.P., Lamb D.H., Heller R., Pietrobon P. (2000). Quantitation of Low Level Unconjugated Polysaccharide in Tetanus Toxoid-Conjugate Vaccine by HPAEC/PAD Following Rapid Separation by Deoxycholate/HCl. J. Pharm. Biomed. Anal..

[31] Gallagher D.T., Oganesyan N., Lees A. (2023). Monomeric Crystal Structure of the Vaccine Carrier Protein CRM_197_ and Implications for Vaccine Development. Acta Crystallogr. F Struct. Biol. Commun..

[32] Elberse K.E.M., Tcherniaeva I., Berbers G.A.M., Schouls L.M. (2010). Optimization and Application of a Multiplex Bead-Based Assay to Quantify Serotype-Specific IgG against *Streptococcus Pneumoniae* Polysaccharides: Response to the Booster Vaccine after Immunization with the Pneumococcal 7-Valent Conjugate Vaccine. Clin. Vaccine Immunol..

[33] Ravenscroft N., Berti F., Rauter A.P., Christensen B.E., Somsák L., Kosma P., Adamo R. (2020). NMR Characterization of Bacterial Glycans and Glycoconjugate Vaccines. Recent Trends in Carbohydrate Chemistry.

[34] WHO Recommendations to Assure the Quality, Safety and Efficacy of Pneumococcal Conjugate Vaccines, Annex 3, TRS No 977. https://www.who.int/publications/m/item/pneumococcal-conjugate-vaccines-annex3-trs-977.

[35] Edmond K.M., Kortsalioudaki C., Scott S., Schrag S.J., Zaidi A.K., Cousens S., Heath P.T. (2012). Group B Streptococcal Disease in Infants Aged Younger than 3 Months: Systematic Review and Meta-Analysis. Lancet.

[36] Madhi S.A., Izu A., Kwatra G., Jones S., Dangor Z., Wadula J., Moultrie A., Adam Y., Pu W., Henry O. (2021). Association of Group B Streptococcus (GBS) Serum Serotype-Specific Anticapsular Immunoglobulin G Concentration and Risk Reduction for Invasive GBS Disease in South African Infants: An Observational Birth-Cohort, Matched Case-Control Study. Clin. Infect. Dis..

[37] Etti M., Calvert A., Galiza E., Lim S., Khalil A., Le Doare K., Heath P.T. (2022). Maternal Vaccination: A Review of Current Evidence and Recommendations. Am. J. Obstet. Gynecol..

[38] Smith W.B., Seger W., Chawana R., Skogeby Z., Silmon De Monerri N.C., Feng Y., Gaylord M., Jongihlati B., Beeslaar J., Skinner J.M. (2025). A Phase 2b Trial Evaluating the Safety, Tolerability, and Immunogenicity of a 6-Valent Group B *Streptococcus* Vaccine Administered Concomitantly With Tetanus, Diphtheria, and Acellular Pertussis Vaccine in Healthy Nonpregnant Female Individuals. J. Infect. Dis..

[39] Bianchi-Jassir F., Paul P., To K.-N., Carreras-Abad C., Seale A.C., Jauneikaite E., Madhi S.A., Russell N.J., Hall J., Madrid L. (2020). Systematic Review of Group B Streptococcal Capsular Types, Sequence Types and Surface Proteins as Potential Vaccine Candidates. Vaccine.

[40] Alderson M.R., Sethna V., Newhouse L.C., Lamola S., Dhere R. (2021). Development Strategy and Lessons Learned for a 10-Valent Pneumococcal Conjugate Vaccine (*PNEUMOSIL^®^*). Hum. Vaccines Immunother..

[41] Frasch C., Preziosi M.-P., LaForce F.M. (2012). Development of a Group A Meningococcal Conjugate Vaccine, MenAfriVacTM. Hum. Vaccines Immunother..

[42] Huang Q., Li D., Kang A., An W., Fan B., Ma X., Ma G., Su Z., Hu T. (2013). PEG as a Spacer Arm Markedly Increases the Immunogenicity of Meningococcal Group Y Polysaccharide Conjugate Vaccine. J. Control. Release.

[43] Shi D., Beasock D., Fessler A., Szebeni J., Ljubimova J.Y., Afonin K.A., Dobrovolskaia M.A. (2022). To PEGylate or Not to PEGylate: Immunological Properties of Nanomedicine’s Most Popular Component, Poly(Ethylene) Glycol and Its Alternatives. Adv. Drug Deliv. Rev..

[44] Carboni F., Adamo R., Fabbrini M., De Ricco R., Cattaneo V., Brogioni B., Veggi D., Pinto V., Passalacqua I., Oldrini D. (2017). Structure of a Protective Epitope of Group B *Streptococcus* Type III Capsular Polysaccharide. Proc. Natl. Acad. Sci. USA.

[45] Micoli F., Adamo R., Costantino P. (2018). Protein Carriers for Glycoconjugate Vaccines: History, Selection Criteria, Characterization and New Trends. Molecules.

[46] Edwards M.S. (2008). Group B Streptococcal Conjugate Vaccine: A Timely Concept for Which the Time Has Come. Hum. Vaccines.

[47] Le Doare K., Benassi V., Cavaleri M., Enwere G., Giersing B., Goldblatt D., Heath P., Hombach J., Isbrucker R., Karampatsas K. (2025). Clinical and Regulatory Development Strategies for GBS Vaccines Intended for Maternal Immunisation in Low- and Middle-Income Countries. Vaccine.

[48] Dangor Z., Kwatra G., Pawlowski A., Fisher P.B., Izu A., Lala S.G., Johansson-Lindbom B., Madhi S.A. (2023). Association of Infant Rib and Alp1 Surface Protein N-Terminal Domain Immunoglobulin G and Invasive Group B Streptococcal Disease in Young Infants. Vaccine.

[49] Shinefield H.R. (2010). Overview of the Development and Current Use of CRM197 Conjugate Vaccines for Pediatric Use. Vaccine.

[50] Fleming-Dutra K.E., Zauche L.H., Roper L.E., Ellington S.R., Olson C.K., Sharma A.J., Woodworth K.R., Tepper N., Havers F., Oliver S.E. (2023). Safety and Effectiveness of Maternal COVID-19 Vaccines Among Pregnant People and Infants. Obstet. Gynecol. Clin. N. Am..

[51] World Health Organization (2014). Weekly Epidemiological Record, 2014, Vol. 89, 29 [Full Issue]. Wkly. Epidemiol. Rec. = Relev. épidémiologique hebdomadaire.

[52] Absalon J., Segall N., Block S.L., Center K.J., Scully I.L., Giardina P.C., Peterson J., Watson W.J., Gruber W.C., Jansen K.U. (2021). Safety and Immunogenicity of a Novel Hexavalent Group B Streptococcus Conjugate Vaccine in Healthy, Non-Pregnant Adults: A Phase 1/2, Randomised, Placebo-Controlled, Observer-Blinded, Dose-Escalation Trial. Lancet Infect. Dis..

[53] WHO WHO Preferred Product Characteristics for Group B Streptococcus Vaccines. https://www.who.int/publications/i/item/WHO-IVB-17.09.

