# Supplementary material for: Preclinical Immunogenicity of a 6-Valent GBS Glycoconjugate Vaccine from a Repeat-Dose GLP Toxicology Study
Source: Vaccines (Basel). 2025 Sep 5;13(9):952. doi: 10.3390/vaccines13090952 (PMC12474038; doi:10.3390/vaccines13090952)
Supplement: Supplementary file 1 [file vaccines-13-00952-s001.zip › Supplementary Data-vaccines-3820572.pdf]

Preclinical Immunogenicity of a 6-valent GBS glycoconjugate vaccine from a repeat-dose GLP Toxicology study

Supplementary Data-Manuscript ID- 3820572

Table 1. Antigenicity, Antigen content, and Identity of each of the six conjugated GBS capsular polysaccharides in the 6-valent GBS conjugate vaccine (GBS-06) used in GLP Toxicology Study

GBS Serotype	Reported Antigen Concentration (µg/mL)	Target antigen concentration (µg/mL)
Ia	40.4	40.0 ± 20 %
Ib	40.2	
II	35.1	
III	43.2	
V	38.2	
VII	40.3	

Table 1: Antigen content of the six conjugated polysaccharides in GBS-06 vaccine formulation used in the GLP Toxicology study. The antigenicity expressed in terms of antigen content and identity of the conjugates were determined by serotype-specific monoclonal antibody using Multiplex ELISA. The target antigen concentration was 40.0 µg/mL. The reported value for antigenicity is in the specification range for each of the six polysaccharide antigens in the 6-valent vaccine.

Table 2. Functional Antibody responses for a representative batch of GBS-06 vaccine using OPA

Dosing Regimen	GBS Ia	GBS Ib	GBS II	GBS III	GBS V	GBS VII
**5 µg + Adjuvant**	1511	1177	2333	768	1474	1024
**10 µg + Adjuvant**	1977	1165	2900	878	1558	987
**Placebo (0.9% Saline)**	< 8	< 8	< 8	< 8	< 8	< 8

Table 2: Serotype-specific opsonic indices (OIs) in Day 35 sera for adjuvanted hexavalent formulations containing 5 µg per dose and 10 µg per dose for each serotype, respectively. Opsonic indices are defined as the reciprocal of the interpolated dilution of serum that kills 50 % of the target bacteria. The value of < 8 indicates that the lowest dilution of sample tested that failed to achieve 50 % bacterial killing.

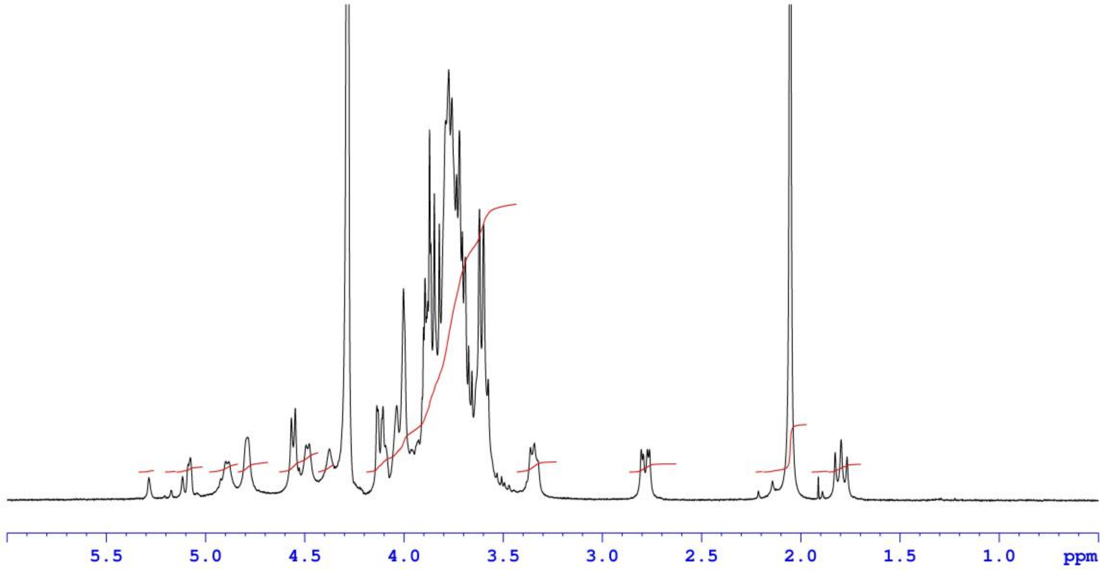

Figure 1. Structural identity GBS Ia PS by 1H-NMR. Data was acquired at 400 MHz frequency at 75 °C and as an average of 40 scans.

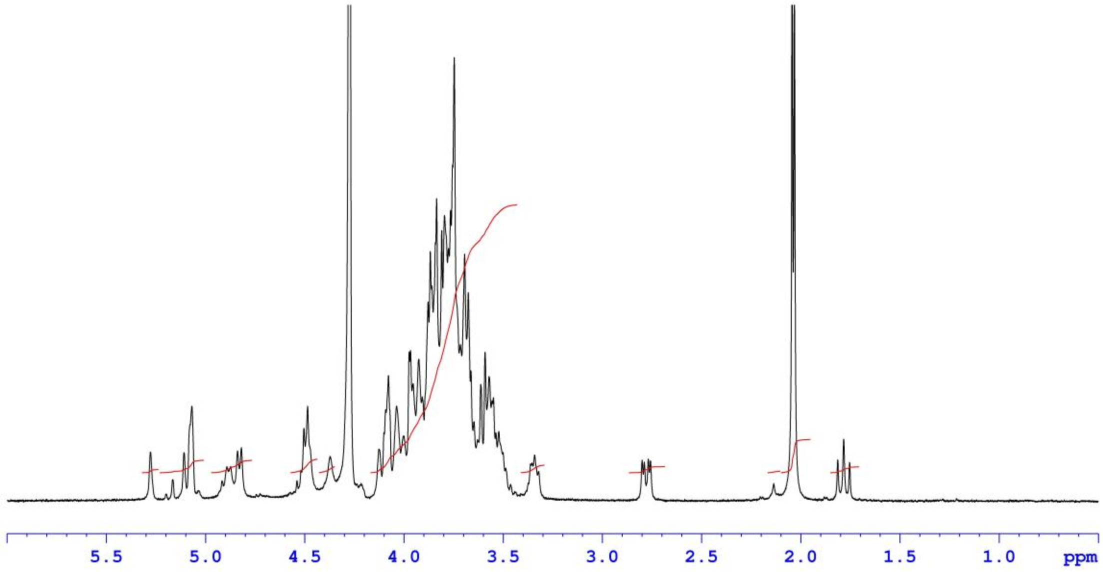

Figure 2. Structural identity GBS Ib PS by 1H-NMR. Data was acquired at 400 MHz frequency at 75 °C and as an average of 40 scans.

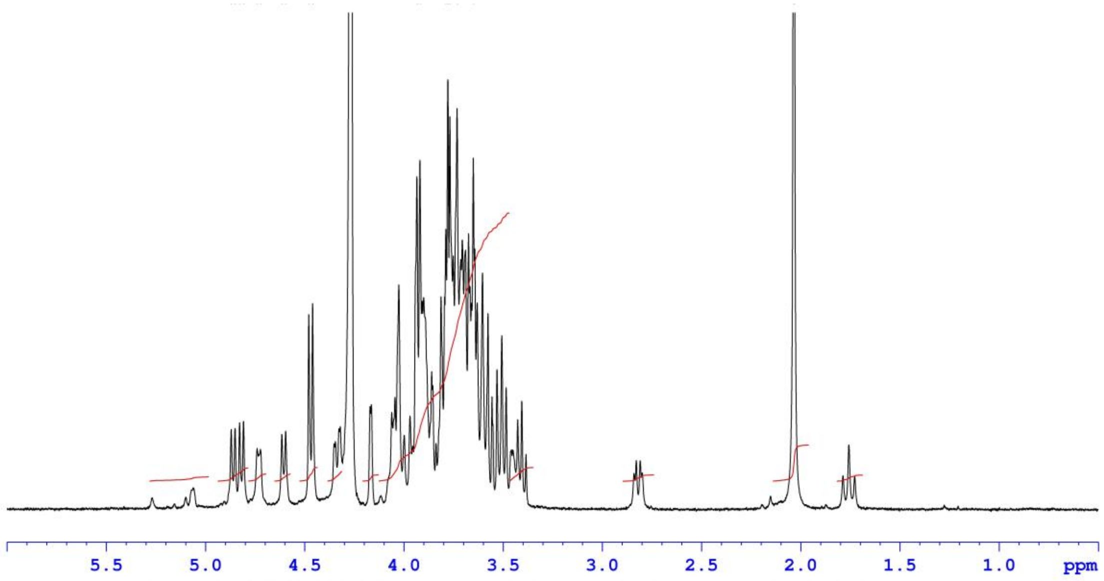

Figure 3. Structural identity GBS II PS by 1H-NMR. Data was acquired at 400 MHz frequency at 75 °C and as an average of 40 scans.

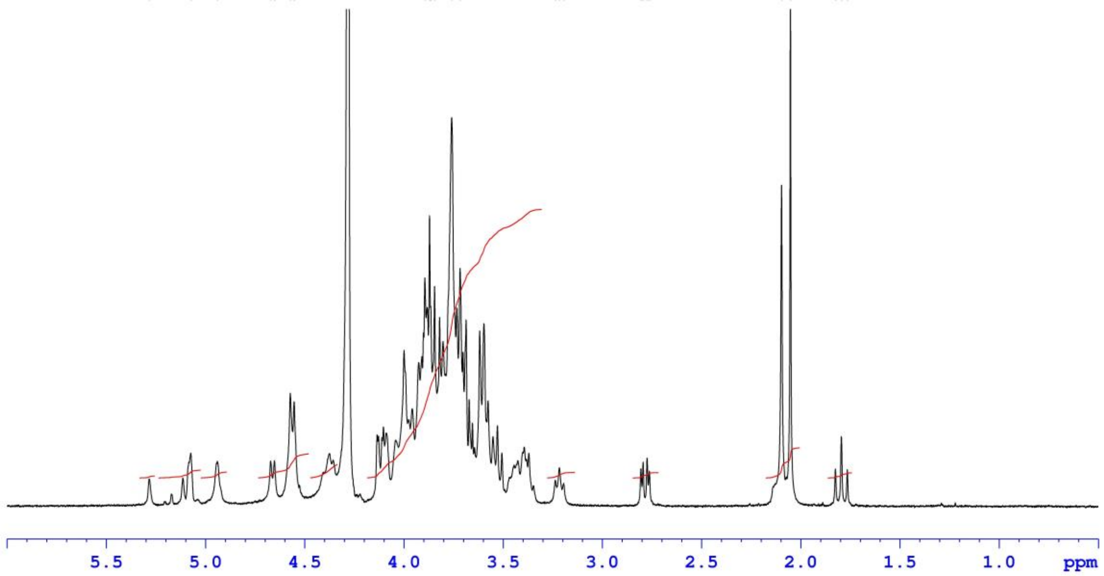

Figure 4. Structural identity GBS V PS by 1H-NMR. Data was acquired at 400 MHz frequency at 75 °C and as an average of 40 scans.

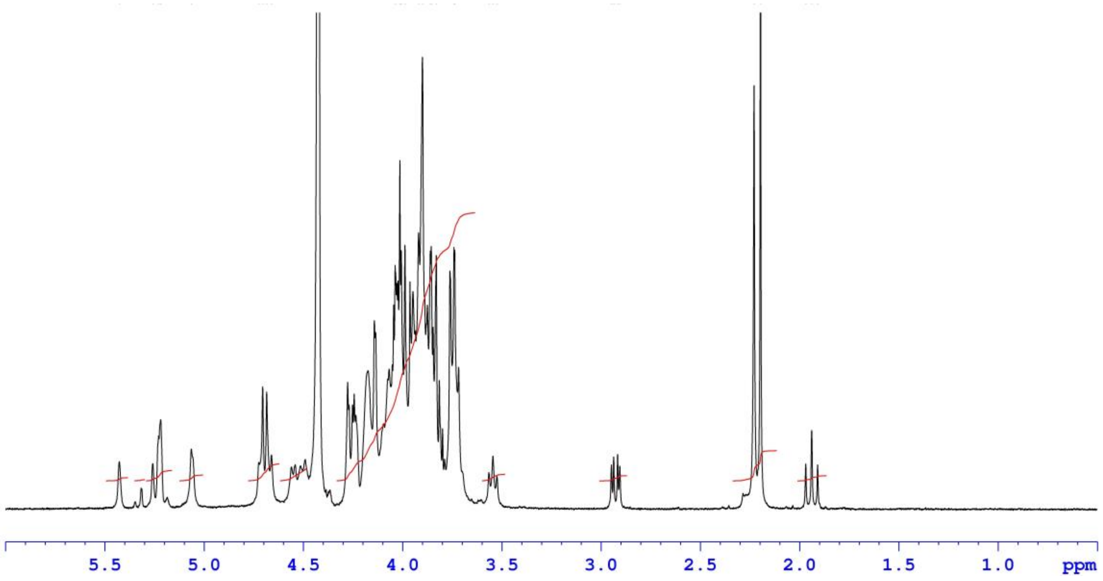

Figure 5. Structural identity GBS VII PS by 1H-NMR. Data was acquired at 400 MHz frequency at 75 °C and as an average of 40 scans.

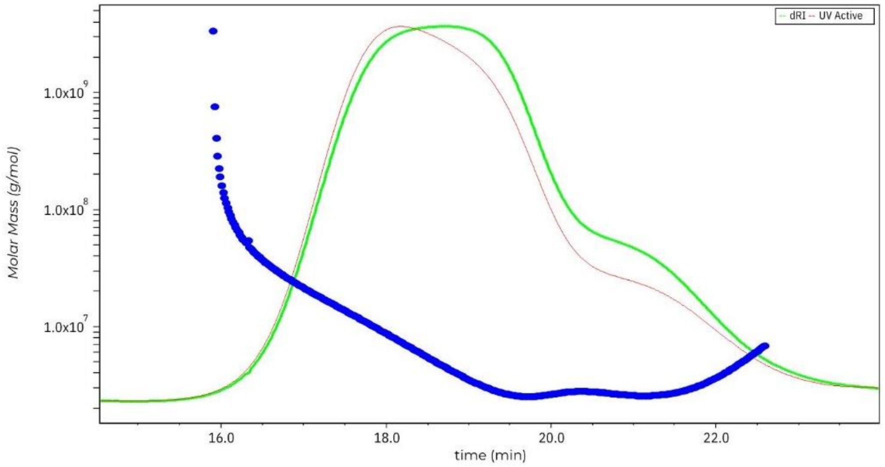

Figure 6. Molar mass vs time graph for GBS Ia monovalent bulk conjugate determined using SEC-MALLS technique. The samples were run on LB-804 and LB-806 columns in tandem at 0.8 mL/min in 1X PBS as mobile phase buffer. Blue, green and red traces represent the conjugate molar mass distribution, Refractive Index (RI) and UV detector signals, respectively.

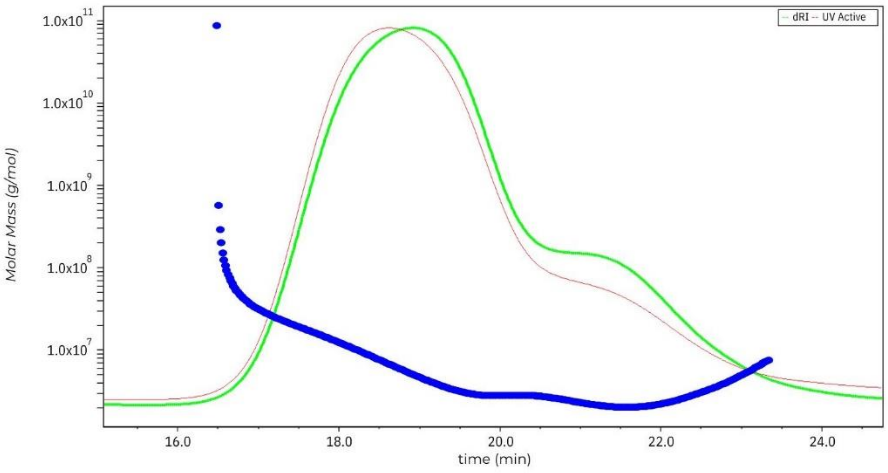

Figure 7. Molar mass vs time graph for GBS Ib monovalent bulk conjugate determined using SEC-MALLS technique. The samples were run on LB-804 and LB-806 columns in tandem at 0.8 mL/min in 1X PBS as mobile phase buffer. Blue, green and red traces represent the conjugate molar mass distribution, Refractive Index (RI) and UV detector signals, respectively.

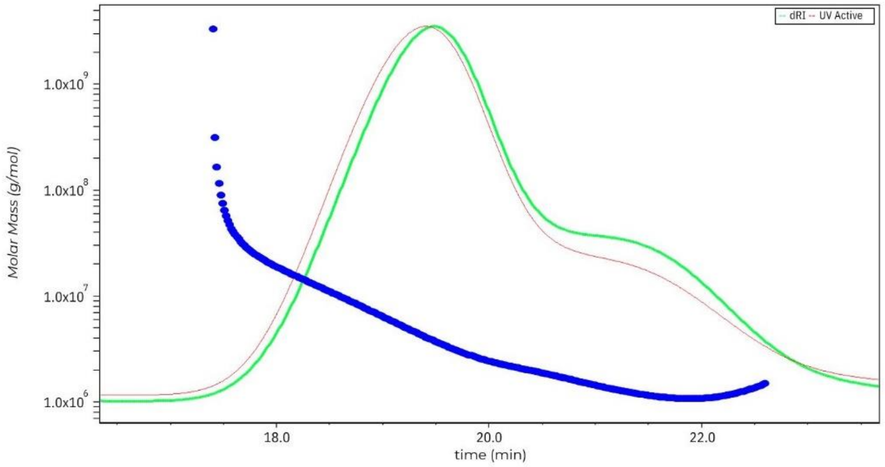

Figure 8. Molar mass vs time graph for GBS II monovalent bulk conjugate determined using SEC-MALLS technique. The samples were run on LB-804 and LB-806 columns in tandem at 0.8 mL/min in 1X PBS as mobile phase buffer. Blue, green and red traces represent the conjugate molar mass distribution, Refractive Index (RI) and UV detector signals, respectively.

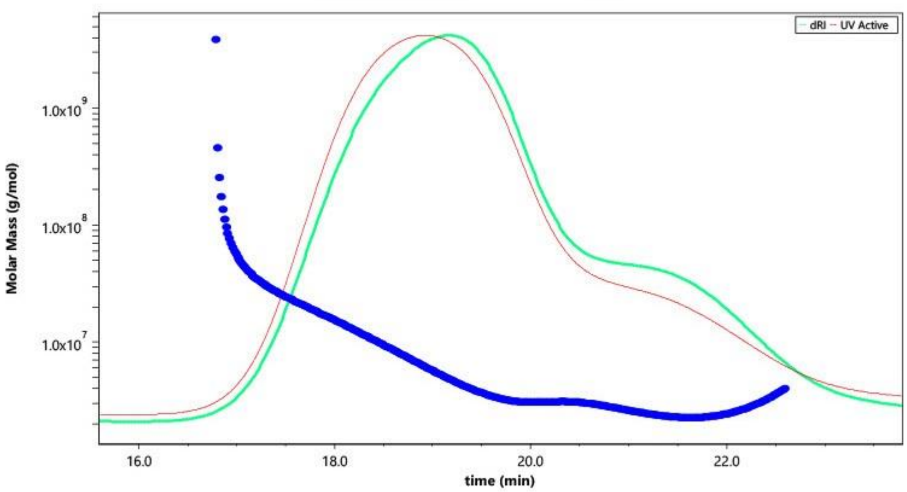

Figure 9. Molar mass vs time graph for GBS III monovalent bulk conjugate determined using SEC-MALLS technique. The samples were run on LB-804 and LB-806 columns in tandem at 0.8 mL/min in 1X PBS as mobile phase buffer. Blue, green and red traces represent the conjugate molar mass distribution, Refractive Index (RI) and UV detector signals, respectively.

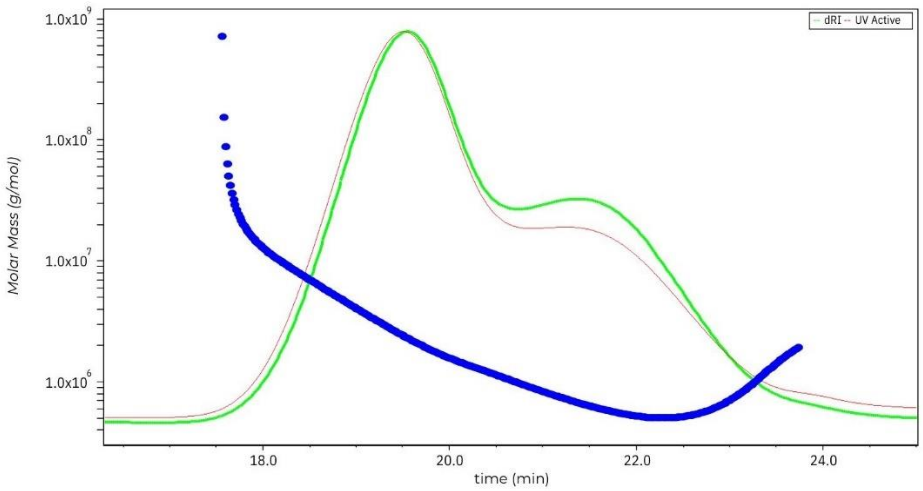

Figure 10. Molar mass vs time graph for GBS V monovalent bulk conjugate determined using SEC-MALLS technique. The samples were run on LB-804 and LB-806 columns in tandem at 0.8 mL/min in 1X PBS as mobile phase buffer. Blue, green and red traces represent the conjugate molar mass distribution, Refractive Index (RI) and UV detector signals, respectively.

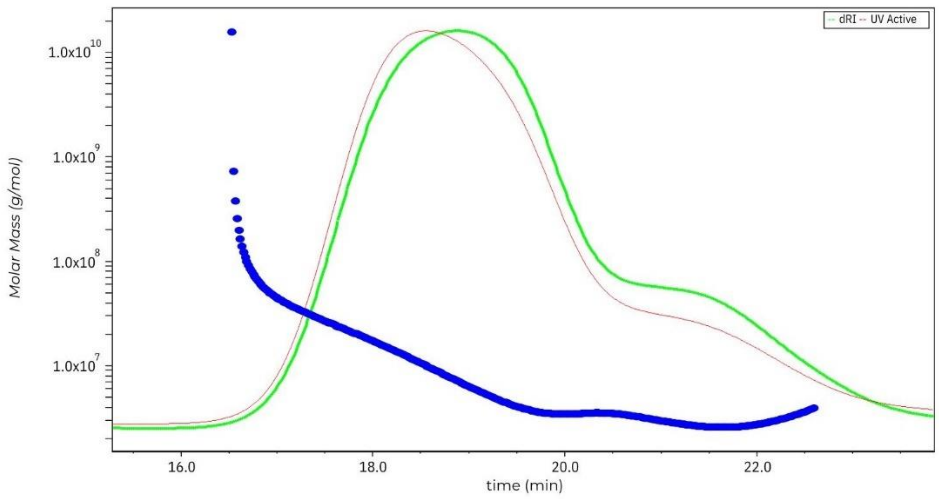

Figure 11. Molar mass vs time graph for GBS VII monovalent bulk conjugate determined using SEC-MALLS technique. The samples were run on LB-804 and LB-806 columns in tandem at 0.8 mL/min in 1X PBS as mobile phase buffer. Blue, green and red traces represent the conjugate molar mass distribution, Refractive Index (RI) and UV detector signals, respectively.

Figure 12. SEC-HPLC profile of derivatized rCRM carrier protein

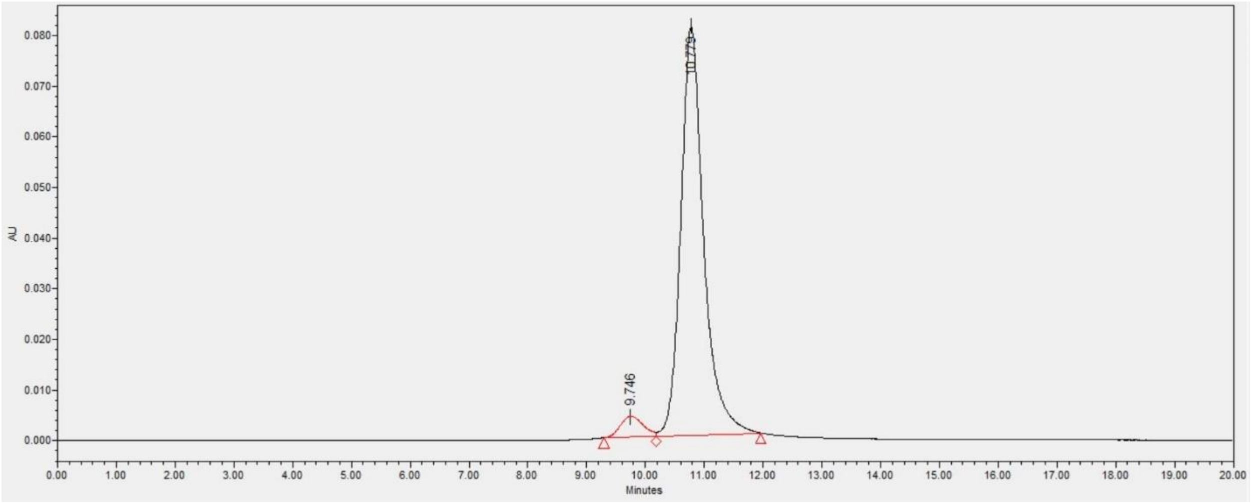

Figure 12: SEC-HPLC profile of a representative batch of derivatized rCRM197. The sample was run using a SEPAK Zenix SEC-150 column at a flow rate of 0.8 mL/min with 1X PBS, pH 7.4 as the mobile phase buffer. The peak at R_t 10.779 min corresponds to the monomer peak of 95.77 % area under the curve. The peak at R_t 9.746 min corresponds to the aggregate peak of 4.23 % area.
