# Supplementary figures and images for: Preclinical Immunogenicity of a 6-Valent GBS Glycoconjugate Vaccine from a Repeat-Dose GLP Toxicology Study
Source: Vaccines (Basel). 2025 Sep 5;13(9):952. doi: 10.3390/vaccines13090952 (PMC12474038; doi:10.3390/vaccines13090952)

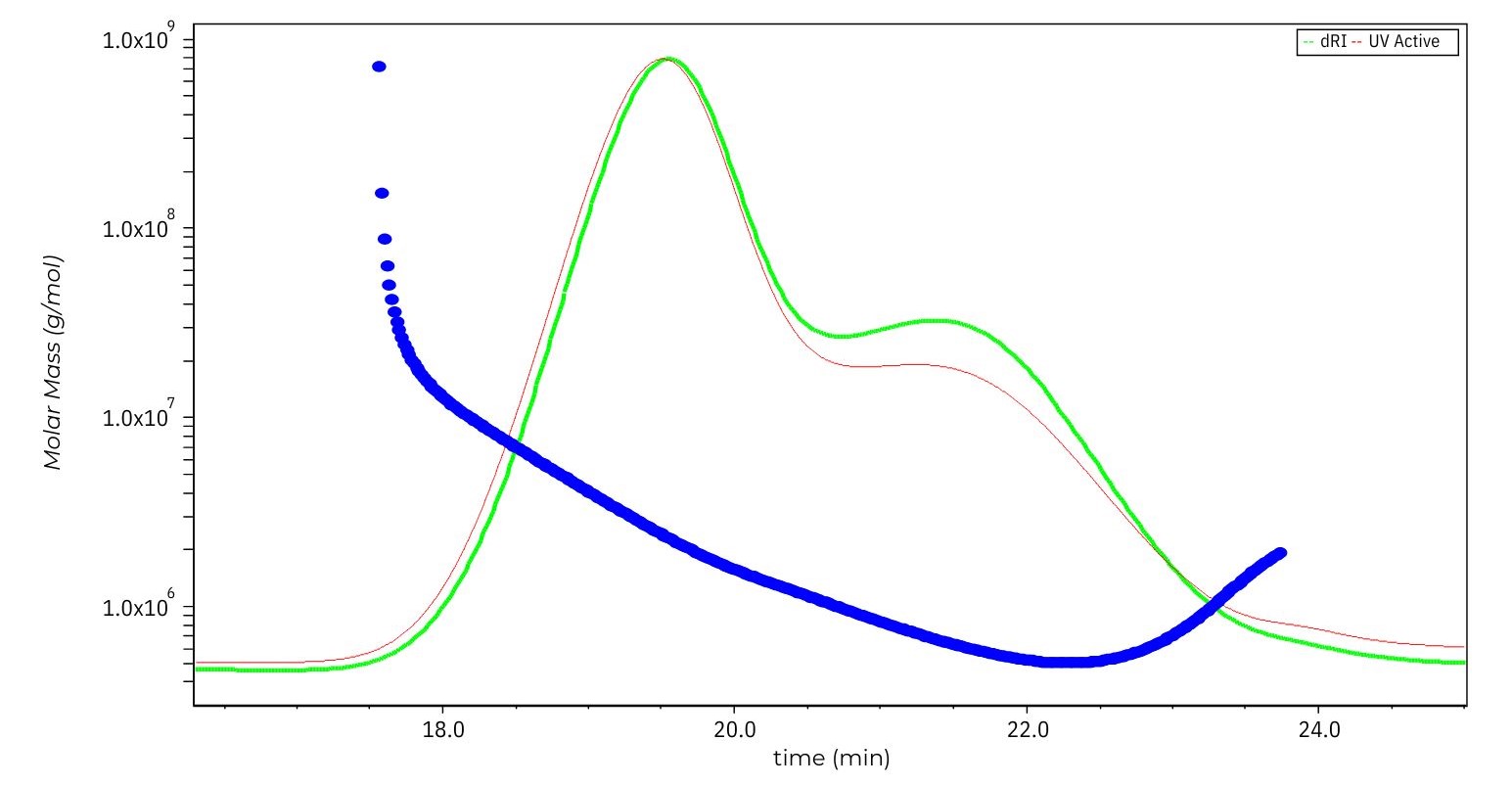

Supplement: Supplementary file 1 [file vaccines-13-00952-s001.zip › Supplementary Figure 10-molar mass exclusion profile of GBS V conjugate.jpg]

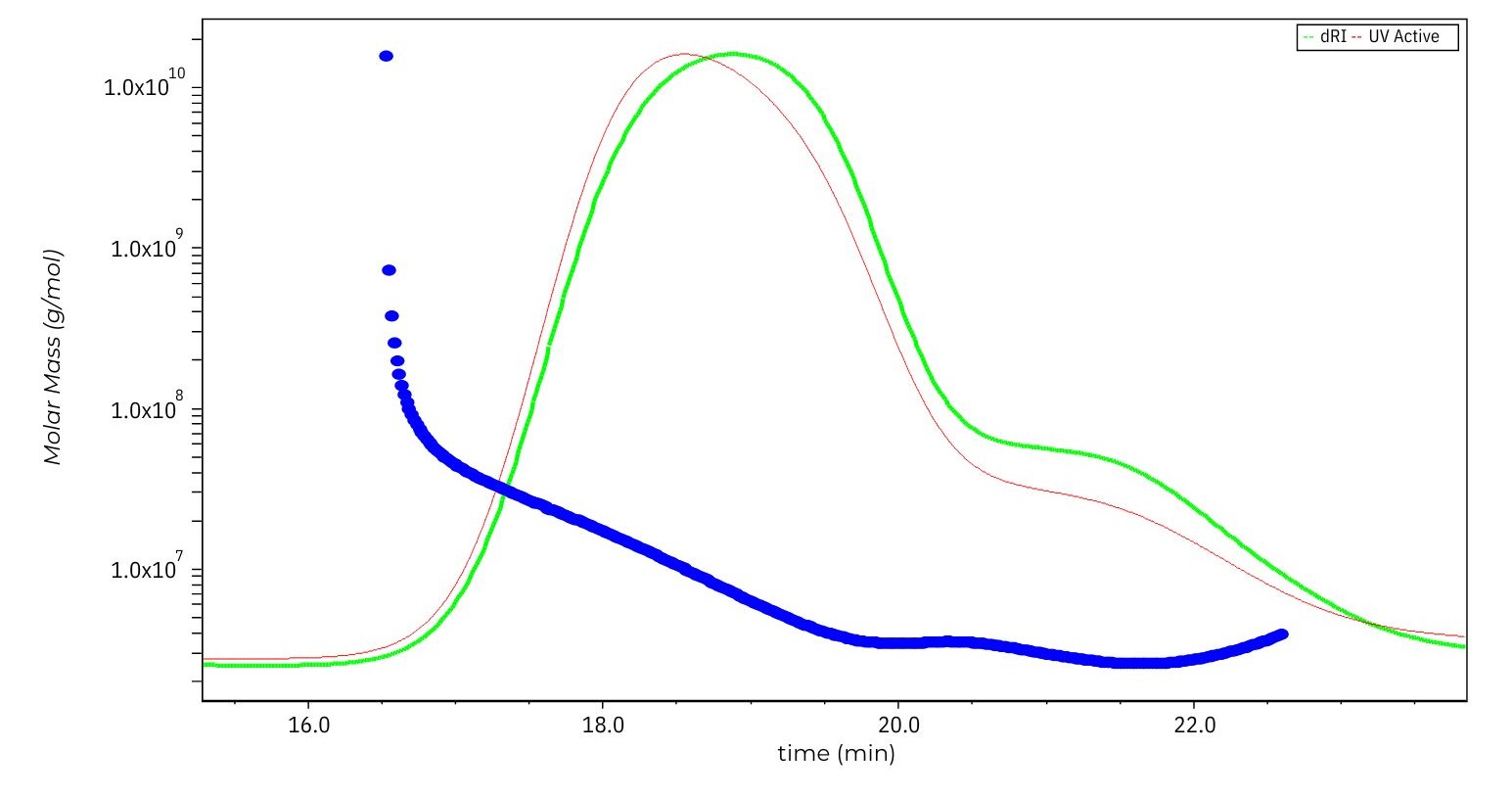

Supplement: Supplementary file 1 [file vaccines-13-00952-s001.zip › Supplementary Figure 11-molar mass exclusion profile of GBS VII conjugate.jpg]

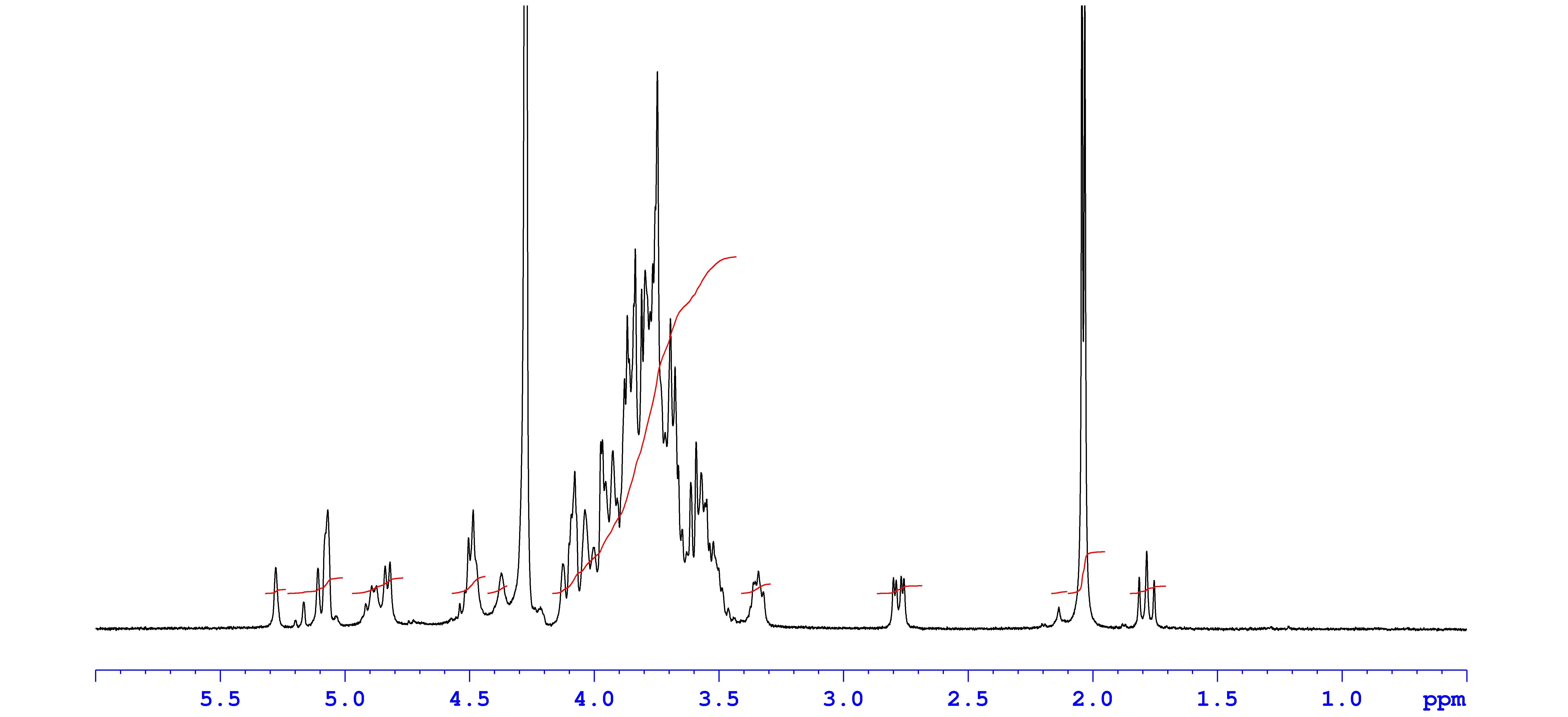

Supplement: Supplementary file 1 [file vaccines-13-00952-s001.zip › Supplementary Figure 2- Proton NMR of GBS Ib PS.jpg]

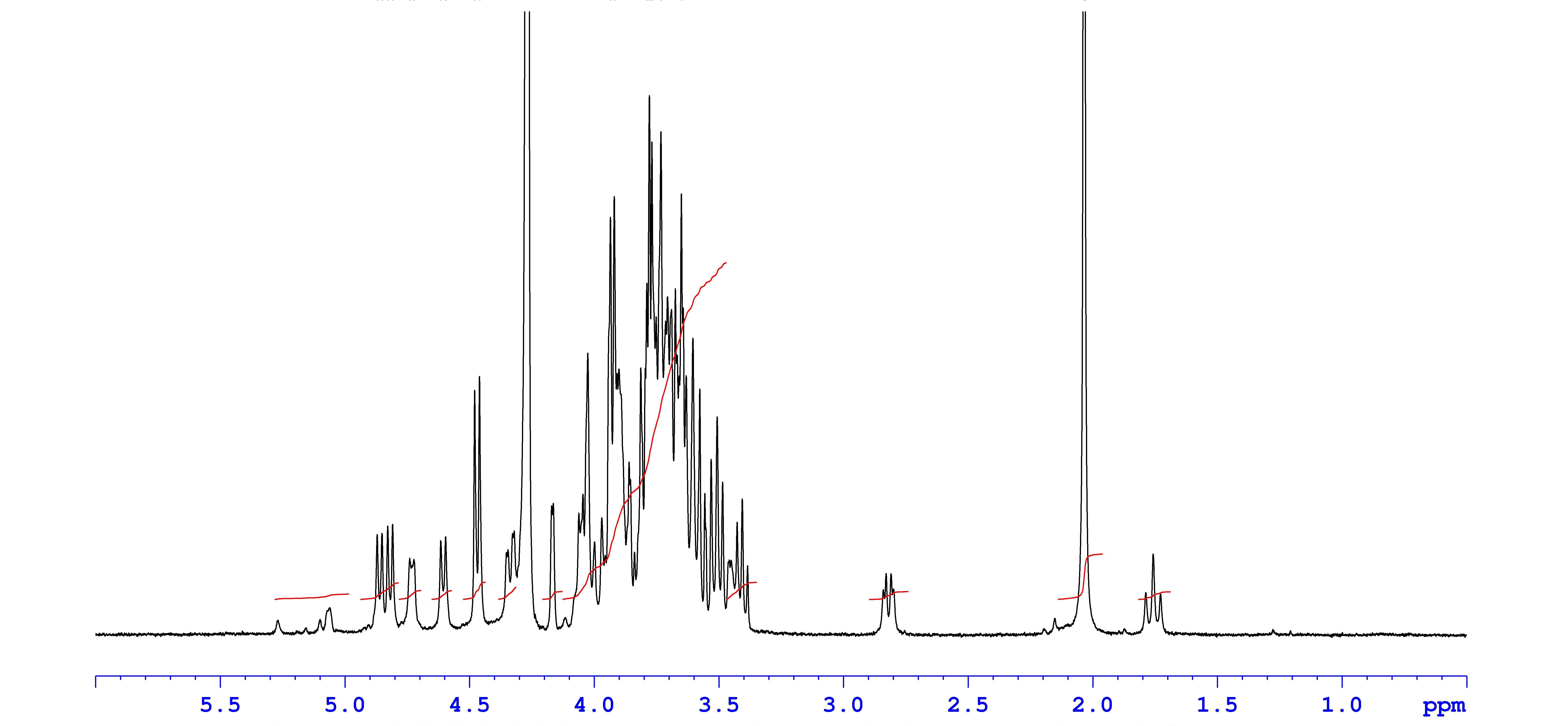

Supplement: Supplementary file 1 [file vaccines-13-00952-s001.zip › Supplementary Figure 3- Proton NMR of GBS II PS.jpg]

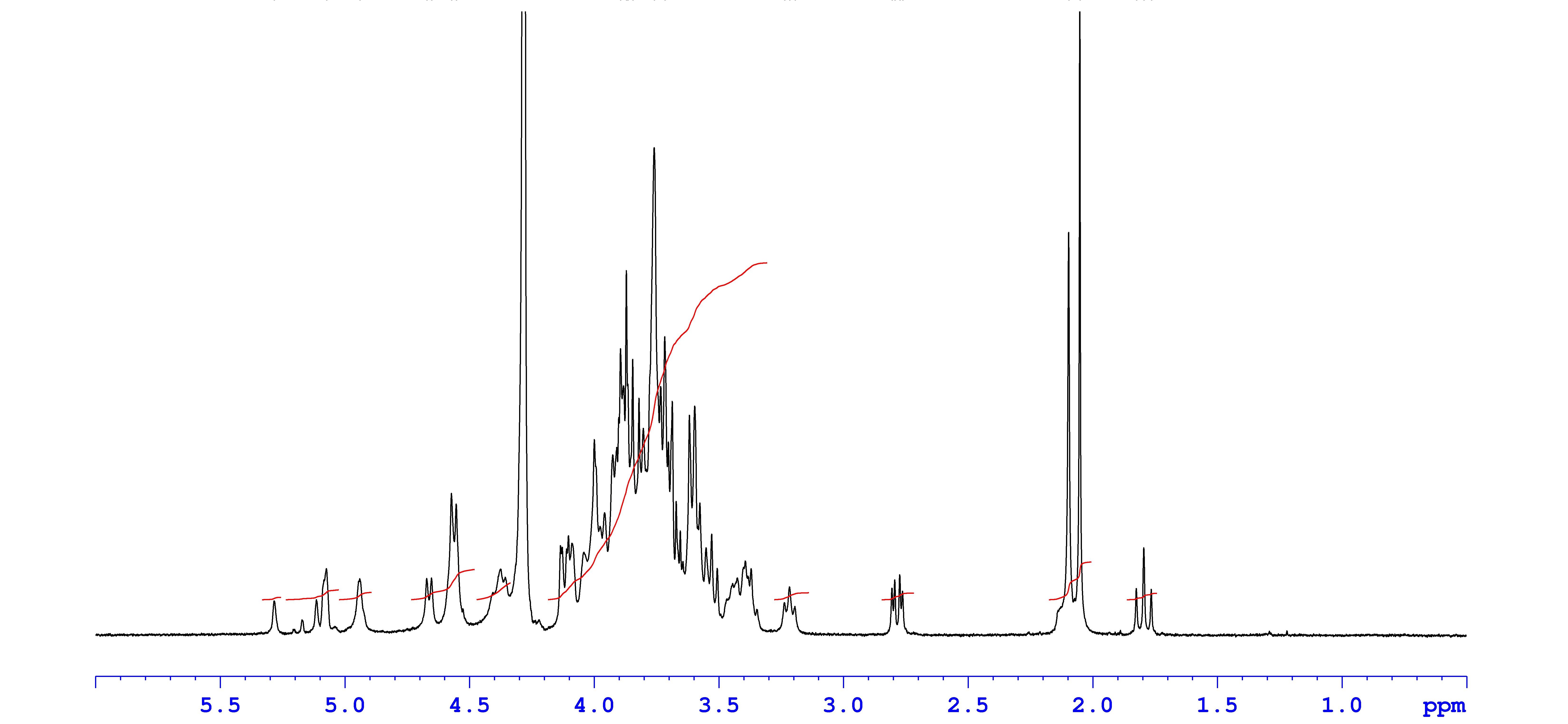

Supplement: Supplementary file 1 [file vaccines-13-00952-s001.zip › Supplementary Figure 4- Proton NMR of GBS V PS.jpg]

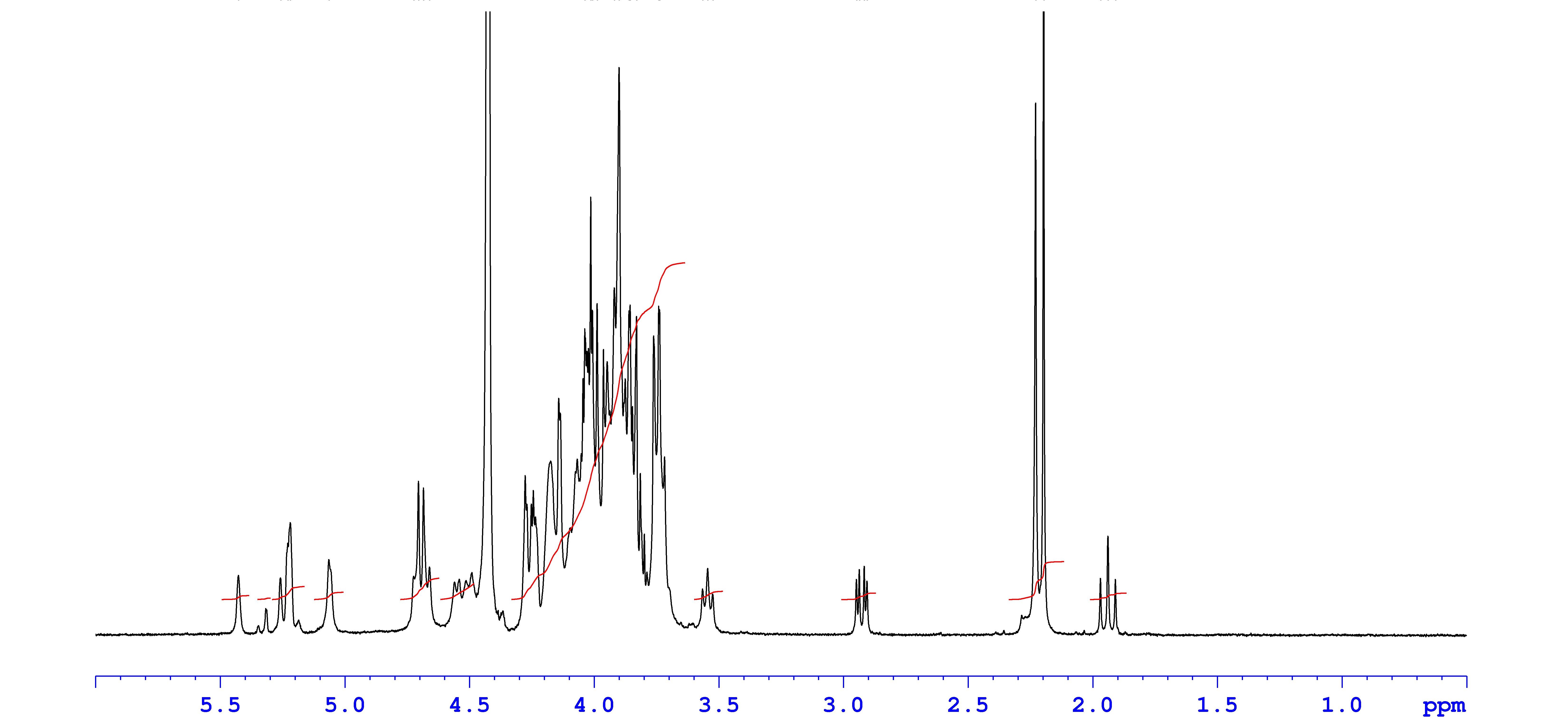

Supplement: Supplementary file 1 [file vaccines-13-00952-s001.zip › Supplementary Figure 5- Proton NMR of GBS VII PS.jpg]

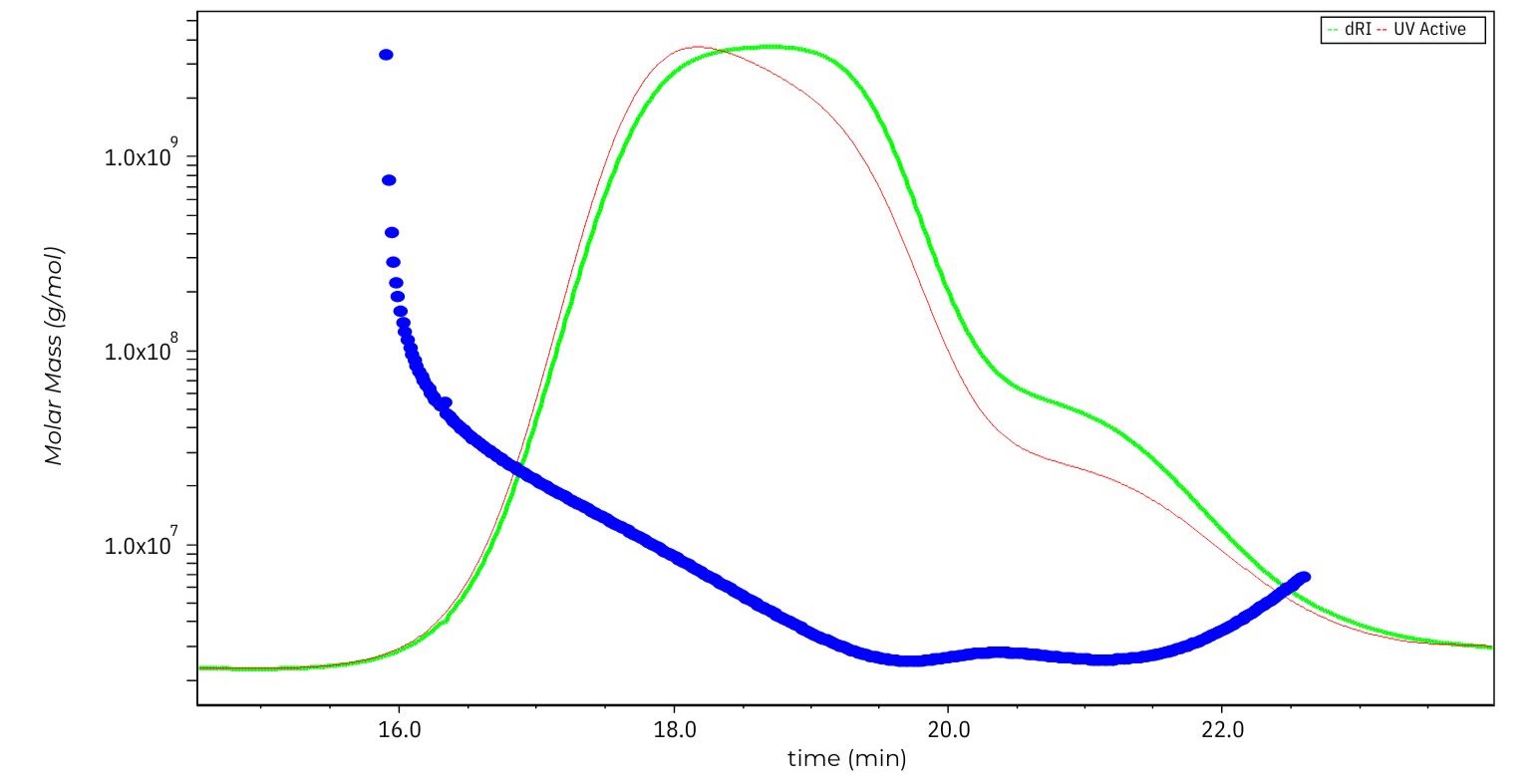

Supplement: Supplementary file 1 [file vaccines-13-00952-s001.zip › Supplementary Figure 6-molar mass exclusion profile of GBS Ia conjugate.jpg]

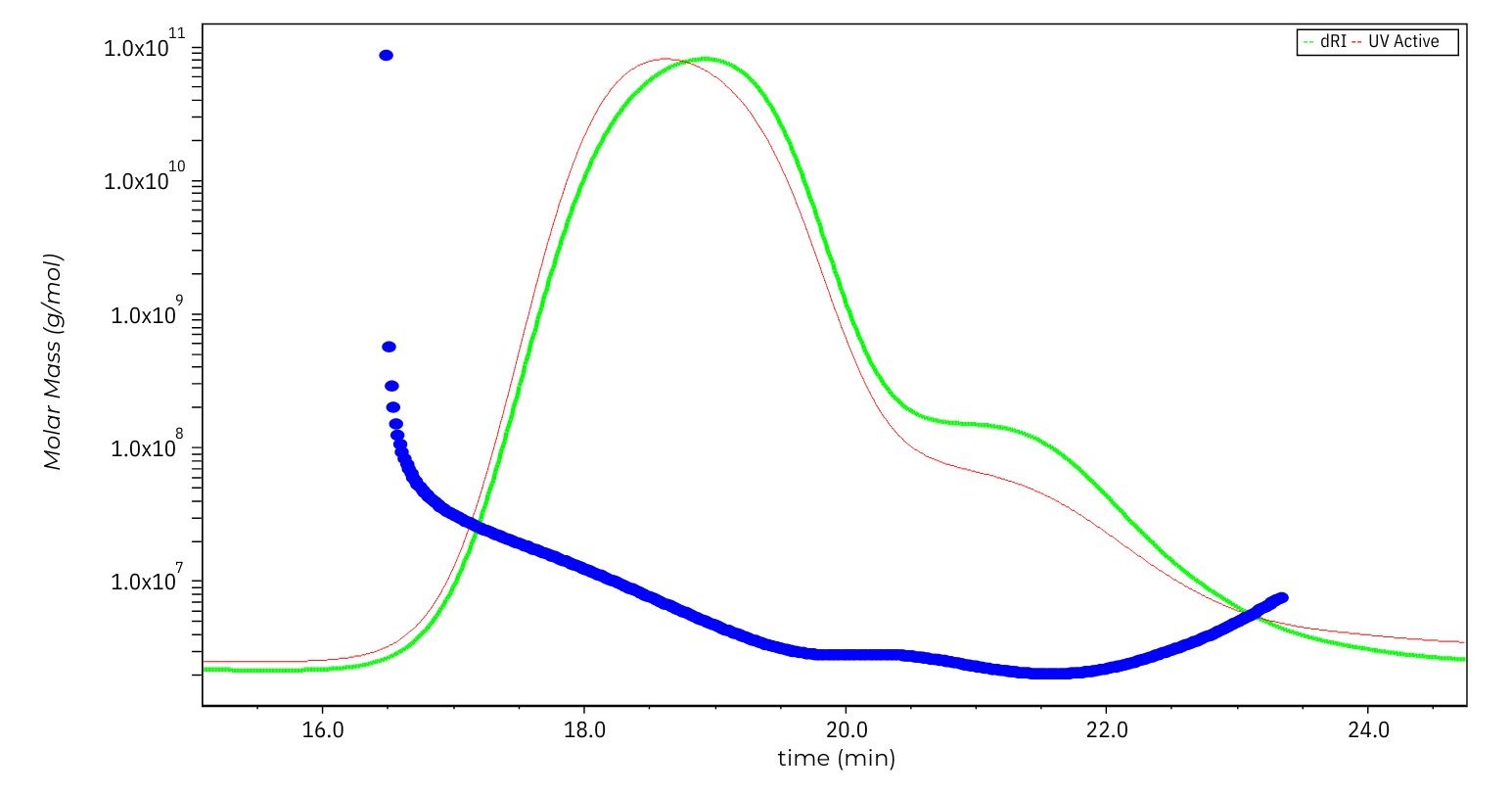

Supplement: Supplementary file 1 [file vaccines-13-00952-s001.zip › Supplementary Figure 7-molar mass exclusion profile of GBS Ib conjugate.jpg]

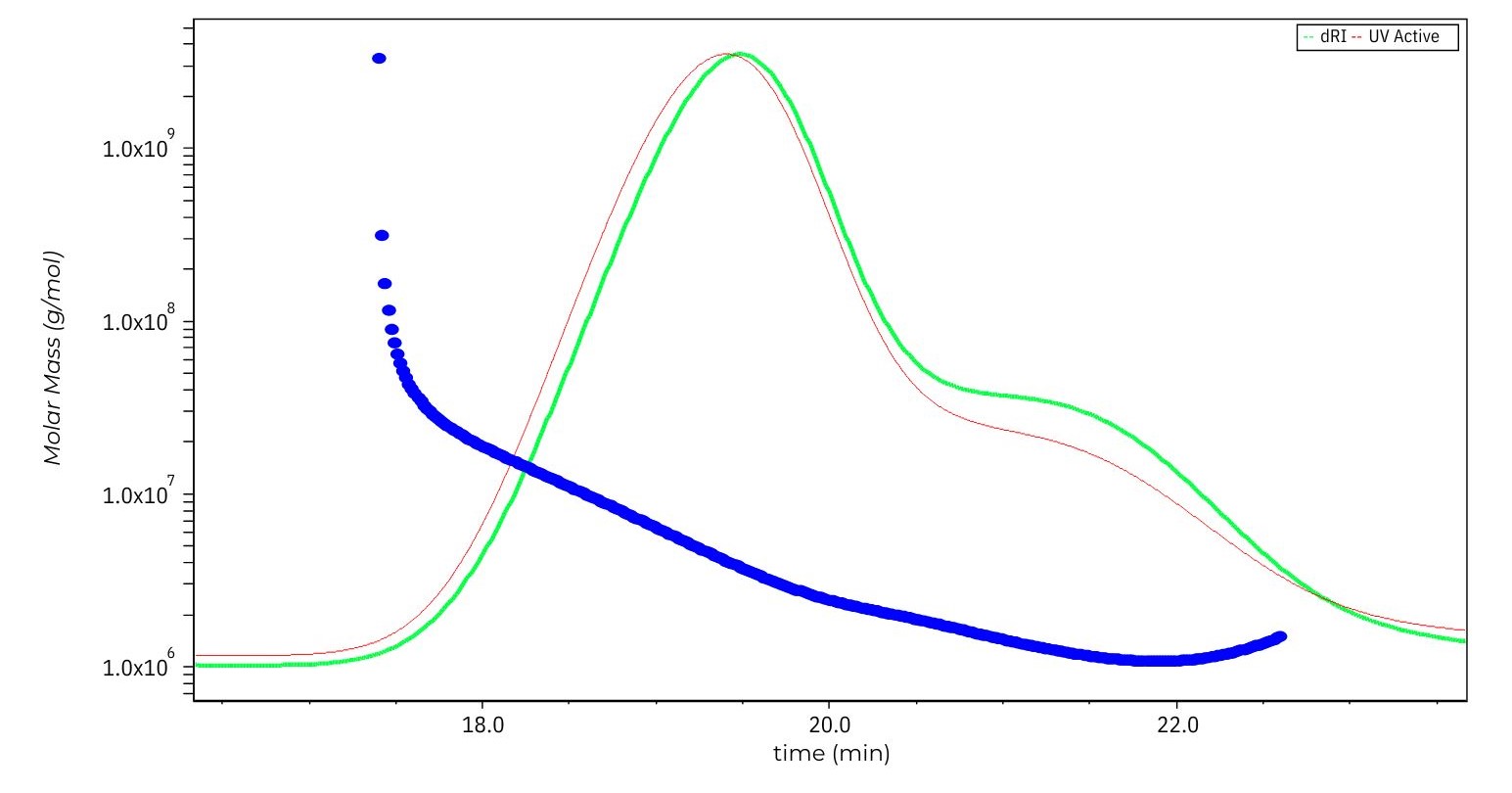

Supplement: Supplementary file 1 [file vaccines-13-00952-s001.zip › Supplementary Figure 8-molar mass exclusion profile of GBS II conjugate.jpg]

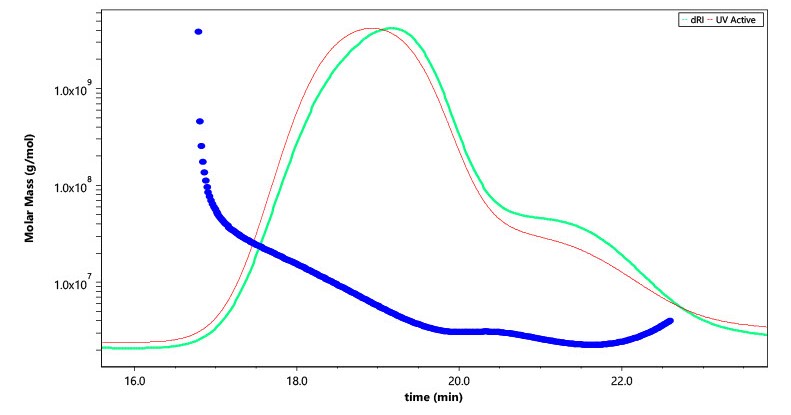

Supplement: Supplementary file 1 [file vaccines-13-00952-s001.zip › Supplementary Figure 9-molar mass exclusion profile of GBS III conjugate.jpg]

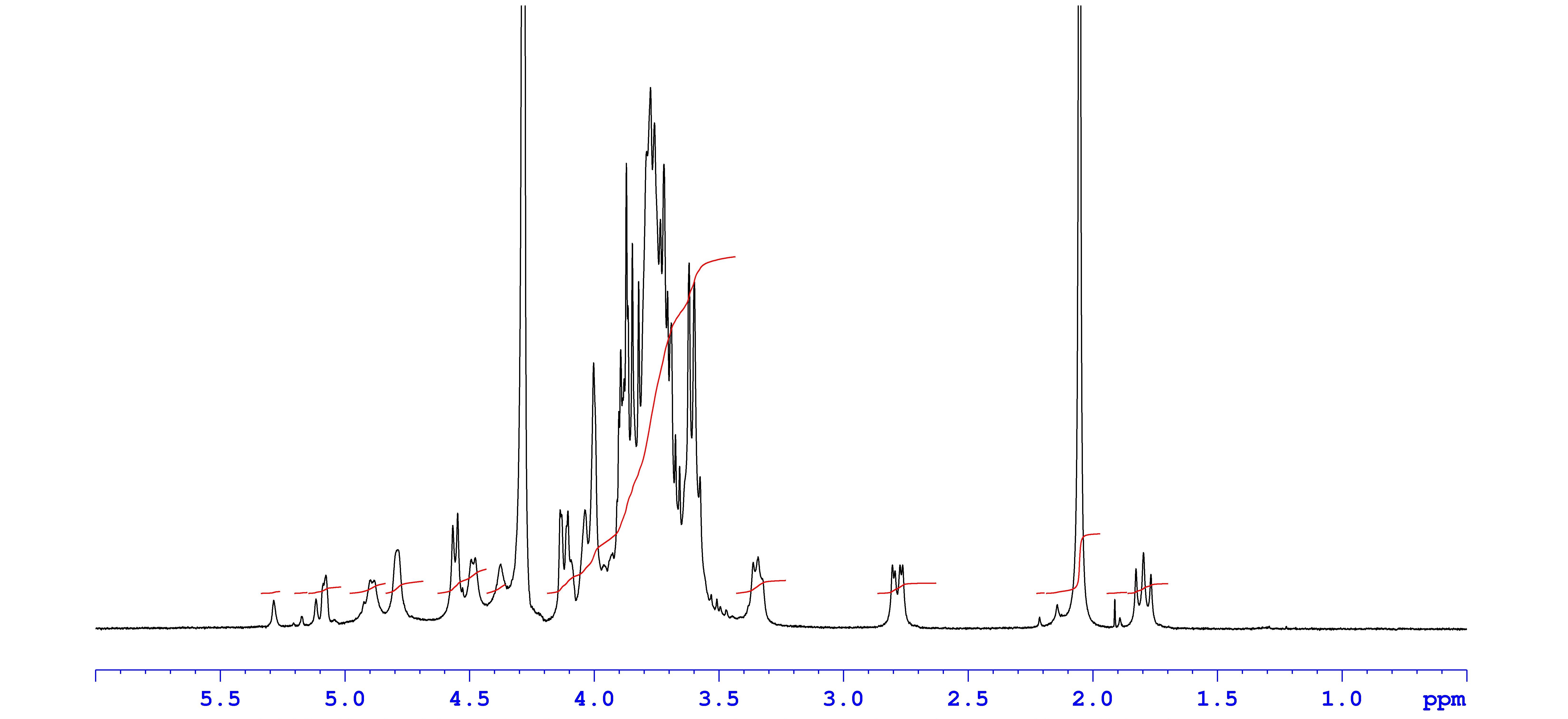

Supplement: Supplementary file 1 [file vaccines-13-00952-s001.zip › Supplementary Figure I- Proton NMR of GBS Ia PS.jpg]
